# Supplementary material for: Effect of surface water and underground water drip irrigation on cotton growth and yield under two different irrigation schemes
Source: PLoS One. 2022 Oct 14;17(10):e0274574. doi: 10.1371/journal.pone.0274574 (PMC9565416; doi:10.1371/journal.pone.0274574)
Supplement: S1 File — (PDF) [file pone.0274574.s004.pdf]

Request for Permission to Publish Content under CC-BY License

Dear Rights Holder or Representative,

I have submitted a paper for publication in a PLOS journal, and wish to include the content listed below in the paper. I'm hereby requesting your (or your company's or institution's) permission to include the content in my paper. Please note that all PLOS journals are publishedunder a Creative Commons Attribution License (CC BY), which allows for unrestricted use and distribution, even commercial, as long as attribution is given to the creator or rights holder of the content. See https://creativecommons.org/licenses/by/4.0/.

To grant me permission to use the content in my PLOS paper, please fill in the information below and then scan the completed form and send it to me at my email address.

Thank you.

My name:

Dr. Cheng Tang

My email address:

tangcheng1983@163.com

Description of the content which I'm seeking permission to use (citation and/or title, andpasted screen shot, if applicable):

The pictures included in "Supplementary figures" file which I have taken by myself in the research field area.

Link to the Content:

Supplementary figures

* * *

On behalf of myself or the rights holder, I hereby grant the permission sought herein.
Signature of Party Granting Permission:

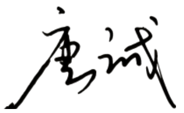

Date:

September 3, 2022

Printed Name and Title:

Cheng Tang/ Professor
