# Supplementary material for: Effect of surface water and underground water drip irrigation on cotton growth and yield under two different irrigation schemes
Source: PLoS One. 2022 Oct 14;17(10):e0274574. doi: 10.1371/journal.pone.0274574 (PMC9565416; doi:10.1371/journal.pone.0274574)
Supplement: Attachment — Submitted filename: PONE-D-22-03631_R1 (1).pdf [file pone.0274574.s007.pdf]

PLOS ONE

Effect of surface water and underground water drip irrigation on cotton growth and yield under two different irrigation schemes

--Manuscript Draft--

**Manuscript Number:**	PONE-D-22-03631R1
**Article Type:**	Research Article
**Full Title:**	Effect of surface water and underground water drip irrigation on cotton growth and yield under two different irrigation schemes
**Short Title:**	Effect of surface and underground water irrigation on Cotton yield by applying different irrigation strategies
**Corresponding Author:**	Cheng Tang, PhD<br>Shihezi University<br>Chengdu, Xinjiang Province CHINA
**Keywords:**	Irrigation strategies; Gossypium hirsutum L; dry matter accumulation; N, P, K uptake; underground water.
**Abstract:**	Two field experiments were conducted to investigate the effects of surface water and underground water drip irrigation on cotton yield, dry matter accumulation, and nutrients uptake under different irrigation regimes. The first experiment comprised of five ratios of underground water to surface water including; 1:0 (U), 0:1 (S), 1:1 (T_3), 1:2 (T_4) and 1:3 (T_5). While, the second experiment comprised of eight treatments including: 1:3 (T_1), 2:2 (T_2), 3:1 (T_3), 1:3 {S:U (T_4)}, 2:2 {S:U (T_5)}, 3:1 {S:U (T_6)}, 4:0 (T_7) and 0:4 (T_8). The average concentration of leaves dry matter after 8th irrigation significantly increased by 131.2% (S), 34.4% (U: S= 1:1), 59.3% (U: S= 1:2), and 93.7% (U: S= 1:3), respectively, compared with U treatment. Likewise, the stem dry matter increased from 48.5 g (U), to 122.2 g (S) and 101.6 g (U:S= 1:3). Furthermore, the results of two-way ANOVAs revealed a significant main and interactive effect on dry matter accumulation of cotton plants treated with surface water at growth, boll and boll opening stages ($p < 0.05$). For instance, the highest leaves dry matter in round irrigation at various growth stages were recorded in T8 treatment. Compared with T7 treatment an average increase rate of 50.6 % (growth), 100.9 % (boll) and 93.3 % (boll opening), in stem dry matter were recorded in T8 treatment. The soil available N at 0-20 cm after 8th irrigation recorded an average increase rate of 40.1%, 6.6%, 13.5%, and 29.5%, respectively, while at 20-40cm an average increase rate of 37.4 %, 7.1%, 20.0%, and 21.9% were noted ($p < 0.05$). Moreover, the concentration of N in round irrigation at 0–20 cm at different growth stages were 83.3 mg/kg (growth stage), 79.01 mg/kg (boll stage), 96.16 mg/kg (boll opening stage) in T8, while in T7 the concentration of N was 36.1 mg/kg (growth), 54.51 mg/kg (boll), and 53.9 mg/kg (boll opening) ($p < 0.05$). The highest cotton yield of 6571 kg/hm^2 was observed in S treatment compared with the U treatment (5492 kg/hm^2, U: S= 1:1 (5502 kg/hm^2), U: S= 1:2 (5873 kg/hm^2) and U: S= 1:3 (6111 kg/hm^2). Similarly, in round irrigation the increasing trend follow the order of T8 > T1 > T4 > T2 > T5 > T3 > T6 > T7. Overall, our findings provide useful information to current irrigation practices in water scarce regions. Alternatively, improving water use efficiency is a viable solution to the water scarcity. Therefore, surface water irrigation is recommended as an effective irrigation strategies to improve cotton yield and growth.
**Order of Authors:**	Nihal Niaz, Master Student
	Cheng Tang, Doctrate
**Opposed Reviewers:**	
**Response to Reviewers:**	Dear Prof. Dr. Rafiq Islam<br>Academic Editor<br>PLOS ONE,<br>Many thanks for your letter. We greatly appreciate the constructive suggestions and comments on our MS entitled “Effect of surface water and underground water drip irrigation on Cotton growth and yield under two different irrigation schemes" from both

Powered by Editorial Manager® and ProduXion Manager® from Aries Systems Corporation

	yourself and the reviewers. All those comments are valuable and very helpful for us to improve our manuscript. We extend our great appreciation for taking the time and efforts to provide such insightful guidance. We have taken a complete consideration to all reviewers' comments as well as those suggestions from the editor's and have made the corrections one by one in the revised version of our manuscript. The changes in the revised MS are marked in track change model.<br><br>We sincerely hope the revised manuscript will be able to meet the requirement and will be finally accepted to publish on your journal of "PLOS ONE". Of course, we are always available to provide ongoing changes to our manuscript if there is any further request either from you or from the reviews.<br>Below please find the revised manuscript and the responses. Again, thank you and all the reviewers for your kinder assistance and we are looking forward to hearing from you at your earliest convenience.<br>Best wishes<br>Many thanks again<br>On behalf of the all coauthors<br>Sincerely yours,
Additional Information:	
**Question**	**Response**
**Financial Disclosure**<br><br>Enter a financial disclosure statement that describes the sources of funding for the work included in this submission. Review the submission guidelines for detailed requirements. View published research articles from *PLOS ONE* for specific examples.<br><br>This statement is required for submission and **will appear in the published article** if the submission is accepted. Please make sure it is accurate.<br><br>**Unfunded studies**<br>Enter: *The author(s) received no specific funding for this work.*<br><br>**Funded studies**<br>Enter a statement with the following details:<br>• Initials of the authors who received each award<br>• Grant numbers awarded to each author<br>• The full name of each funder<br>• URL of each funder website<br>• Did the sponsors or funders play any role in the study design, data collection and analysis, decision to publish, or preparation of the manuscript?<br>• **NO** - Include this sentence at the end of your statement: *The funders had no role in study design, data collection and analysis, decision to publish, or preparation of the manuscript.*<br>• **YES** - Specify the role(s) played.	No

* typeset	
**Competing Interests**<br><br>Use the instructions below to enter a competing interest statement for this submission. On behalf of all authors, disclose any competing interests that could be perceived to bias this work—acknowledging all financial support and any other relevant financial or non-financial competing interests.<br><br>This statement is required for submission and **will appear in the published article** if the submission is accepted. Please make sure it is accurate and that any funding sources listed in your Funding Information later in the submission form are also declared in your Financial Disclosure statement.<br><br>View published research articles from *PLOS ONE* for specific examples.<br><br>**NO authors have competing interests**<br><br>Enter: *The authors have declared that no competing interests exist.*<br><br>**Authors with competing interests**<br><br>Enter competing interest details beginning with this statement:<br><br>*I have read the journal's policy and the authors of this manuscript have the following competing interests: [insert competing interests here]*<br><br>* typeset	The authors have declared that no competing interests exist.
**Ethics Statement**<br><br>Enter an ethics statement for this submission. This statement is required if the study involved:	N/A

- Human participants
- Human specimens or tissue
- Vertebrate animals or cephalopods
- Vertebrate embryos or tissues
- Field research

Write "N/A" if the submission does not require an ethics statement.

General guidance is provided below. Consult the submission guidelines for detailed instructions. **Make sure that all information entered here is included in the Methods section of the manuscript.**

Powered by Editorial Manager® and ProduXion Manager® from Aries Systems Corporation

**Format for specific study types**<br><br>**Human Subject Research (involving human participants and/or tissue)**<br>• Give the name of the institutional review board or ethics committee that approved the study<br>• Include the approval number and/or a statement indicating approval of this research<br>• Indicate the form of consent obtained (written/oral) or the reason that consent was not obtained (e.g. the data were analyzed anonymously)<br><br>**Animal Research (involving vertebrate animals, embryos or tissues)**<br>• Provide the name of the Institutional Animal Care and Use Committee (IACUC) or other relevant ethics board that reviewed the study protocol, and indicate whether they approved this research or granted a formal waiver of ethical approval<br>• Include an approval number if one was obtained<br>• If the study involved *non-human primates*, add *additional details* about animal welfare and steps taken to ameliorate suffering<br>• If anesthesia, euthanasia, or any kind of animal sacrifice is part of the study, include briefly which substances and/or methods were applied<br><br>**Field Research**<br><br>Include the following details if this study involves the collection of plant, animal, or other materials from a natural setting:<br>• Field permit number<br>• Name of the institution or relevant body that granted permission	
**Data Availability**<br><br>Authors are required to make all data underlying the findings described fully available, without restriction, and from the time of publication. PLOS allows rare exceptions to address legal and ethical concerns. See the PLOS Data Policy and FAQ for detailed information.	Yes - all data are fully available without restriction

A Data Availability Statement describing where the data can be found is required at submission. Your answers to this question constitute the Data Availability Statement and **will be published in the article**, if accepted.<br><br>**Important:** Stating 'data available on request from the author' is not sufficient. If your data are only available upon request, select 'No' for the first question and explain your exceptional situation in the text box.<br><br>Do the authors confirm that all data underlying the findings described in their manuscript are fully available without restriction?	
**Describe where the data may be found in full sentences. If you are copying our sample text, replace any instances of XXX with the appropriate details.**<br><br>• If the data are **held or will be held in a public repository**, include URLs, accession numbers or DOIs. If this information will only be available after acceptance, indicate this by ticking the box below. For example: *All XXX files are available from the XXX database (accession number(s) XXX, XXX.)*.<br>• If the data are all contained **within the manuscript and/or Supporting Information files**, enter the following: *All relevant data are within the manuscript and its Supporting Information files.*<br>• If neither of these applies but you are able to provide **details of access elsewhere**, with or without limitations, please do so. For example:<br><br>*Data cannot be shared publicly because of [XXX]. Data are available from the XXX Institutional Data Access / Ethics Committee (contact via XXX) for researchers who meet the criteria for access to confidential data.*<br><br>*The data underlying the results presented in the study are available from (include the name of the third party*	All relevant data are within the manuscript and its supporting information files (Zip file).

and contact information or URL).<br>• This text is appropriate if the data are owned by a third party and authors do not have permission to share the data.<br><br>* typeset	
Additional data availability information:	Tick here if the URLs/accession numbers/DOIs will be available only after acceptance of the manuscript for publication so that we can ensure their inclusion before publication.

Powered by Editorial Manager® and ProduXion Manager® from Aries Systems Corporation

Cover letter

Dear editor,

Please find enclosed a manuscript titled “*Effect of surface water and underground water drip irrigation on Cotton growth and yield under two different irrigation schemes*” authored by Nihal Niaz and Cheng Tang, for consideration of publication in Journal of “PLOS ONE”. This manuscript included 18 pages, 8 tables and 6 figures. The work described has not been submitted elsewhere for publication, in whole or in part, and all the authors listed have approved the manuscript that is enclosed. We believe the paper may be of particular interest to the readers of your journal as it deals with the Effect of surface water and underground water drip irrigation on Cotton growth and yield under two different irrigation schemes, which could not only enhance our understanding about the mechanism of soil water irrigations managements, also our findings will provide useful technical guidance on how to improve irrigation methods along with fertigation to improve WUE, cotton yield, dry matter accumulation and soil N,P,K status especially in calcareous soil. We believe the paper may be of particular interest to the readers of your journal.

We hope this paper is suitable for journal of “PLOS ONE”. We appreciate your consideration of our manuscript and we look forward to receiving positive comments from the reviewers. Much thanks again for your attention!

Sincerely,

Best wishes

On behalf of all co-authors.

Manuscript Click here to access/download;Manuscript;Manuscript.doc

Type of contribution: Research Article

Number of text pages: 18

Number of tables: 8

Number of figures: 6

Title:

Effect of surface water and underground water drip irrigation on cotton growth and yield under two different irrigation schemes

Nihal Niaz[1] and Cheng Tang[1]*

[1]College of Agronomy, Shihezi University, Shihezi City, 832003, Xinjiang Province, China.

***Correspondence:** tangcheng1983@163.com; Tel.: +86 15352608029

Both authors contributed equally to this work

ABSTRACT: Two field experiments were conducted to investigate the effects of surface water and underground water drip irrigation on cotton yield, dry matter accumulation, and nutrients uptake under different irrigation regimes. The first experiment comprised of five ratios of underground water to surface water including; 1:0 (U), 0:1 (S), 1:1 (T_3), 1:2 (T_4) and 1:3 (T_5). While, the second experiment comprised of eight treatments including: 1:3 (T_1), 2:2 (T_2), 3:1 (T_3), 1:3 {S:U (T_4)}, 2:2 {S:U (T_5)}, 3:1 {S:U (T_6)}, 4:0 (T_7) and 0:4 (T_8). The average concentration of leaves dry matter after 8th irrigation significantly increased by 131.2% (S),

34.4% (U: S= 1:1), 59.3% (U: S= 1:2), and 93.7% (U: S= 1:3), respectively, compared with U treatment. Likewise, the stem dry matter increased from 48.5 g (U), to 122.2 g (S) and 101.6 g (U:S= 1:3). Furthermore, the results of two-way ANOVAs revealed a significant main and interactive effect on dry matter accumulation of cotton plants treated with surface water at growth, boll and boll opening stages ($p < 0.05$). For instance, the highest leaves dry matter in round irrigation at various growth stages were recorded in T8 treatment. Compared with T7 treatment an average increase rate of 50.6 % (growth), 100.9 % (boll) and 93.3 % (boll opening), in stem dry matter were recorded in T8 treatment. The soil available N at 0-20 cm after 8th irrigation recorded an average increase rate of 40.1%, 6.6%, 13.5%, and 29.5%, respectively, while at 20-40cm an average increase rate of 37.4 %, 7.1%, 20.0%, and 21.9% were noted ($p < 0.05$). Moreover, the concentration of N in round irrigation at 0–20 cm at different growth stages were 83.3 mg/kg (growth stage), 79.01 mg/kg (boll stage), 96.16 mg/kg (boll opening stage) in T8, while in T7 the concentration of N was 36.1 mg/kg (growth), 54.51 mg/kg (boll), and 53.9 mg/kg (boll opening) ($p < 0.05$). The highest cotton yield of 6571 kg/hm^2 was observed in S treatment compared with the U treatment (5492 kg/hm^2, U: S= 1:1 (5502 kg/hm^2), U: S= 1:2 (5873 kg/hm^2) and U: S= 1:3 (6111 kg/hm^2). Similarly, in round irrigation the increasing trend follow the order of T8 > T1 > T4 > T2 > T5 > T3 > T6 > T7. Overall, our findings provide useful information to current irrigation practices in water scarce regions. Alternatively, improving water use efficiency is a viable solution to the water scarcity. Therefore, surface water irrigation is recommended as an effective irrigation strategies to improve cotton yield and growth.

Keywords: Irrigation strategies; Gossypium hirsutum L; dry matter accumulation; N, P, K uptake; underground water.

1. Introduction

Water for irrigation is one of the most limiting factors for future global agricultural development [1, 2]. Climate change, and over-exploitation of water resources in arid and semi-arid regions of the world are being subjected to severe water shortages [3]. For instance, , in China the arid and semi-arid area occupies 52.5% of China's land area which is mainly distributed in Northwest, Northeast, and North China [4]. To achieve high grain yields in these regions sufficient irrigation is required, as there are no reliable surface water sources for irrigation, groundwater is the main source of water for irrigation. Moreover, to fulfill the inherent irrigation water requirements, about 60% of the water resources including both surface and underground water is used for agriculture purposes. On the other hand, due to significant increase in population, there is competition for fresh water among municipal, industrial and agricultural sectors in several countries in the world especially in China [5]. Consequently, the allocation of fresh water to agriculture sector in these regions substantially decreased [6]. Therefore, improving irrigation efficiencies by introducing new irrigation strategies is the key and effective way to solve the problem of water shortage, ecological environment deterioration, and to develop agricultural production system in arid and semi-arid regions.

Cotton (*Gossypium hirsutum* L.), an important source of natural fibers for textile industries that serve the humanity from at least more than four to seven thousand years ago [7]. The current global cotton fiber production is estimated to be 24.65 million tons, with 6.71 million tons produced in America, 0.38 million tons produced in Europe, and 15.06 million tons produced in Asia [8]. Following this, only China accounts for one-quarter of the world's cotton output and one-third of the world's cotton consumption [9]. On the flip side, cotton production is completely dependent on irrigation i.e., the shortage of irrigation water resources restricts the

comprehensive improvement of cotton productivity [10, 11]. On average, water requirements for cotton growth vary from 700 to 1200 mm during the growing season, depending on irrigation method, and production goals [12]. Numerous studies have been conducted to examine water-use efficiency of cotton in recent years. For instance, a study conducted by Grismer [13] noted that, in Arizona counties, for upland cotton actual evapotranspiration (ETc) water-use efficiency varied from 1.27 to 1.38 kg/ha-mm while, for pima cotton, its varied from 0.9 to 1.09 kg/ha-mm. In California counties, ETc water-use efficiency varied from 1.34 to 2.10 kg/ha-mm and 1.51–1.77 kg/ha-mm for upland and pima varieties, respectively. Furthermore, due to unevenly distribution of annual precipitation across the season, both surface water resources (water from rivers and reservoirs) and groundwater resources (water stored in aquifers) were used for agriculture purposes in arid and semi-arid regions. However, irrigation with underground water has a negative impact on cotton productivity, plant nutritional condition, and dry matter accumulation. For instance, excessive underground water irrigation exacerbates the soil salinization problems, and reduce crop yield [11, 14]. Likewise, well water irrigation with low temperature potentially inhibits the growth and development of cotton plant [15]. Moreover, low soil temperatures may slow down the uptake rate of nutrients such as N so much that they turn out to limit vegetative growth rates [16]. Following this at 20°C-RZT, nutrient concentrations have been significantly affected plant growth indexes, indicating that low root temperature inhibited high nutrient effects on plant growth [16, 17]. While on the other hand, surface water irrigation shows promising effect on increasing crops yield [18, 19]. For example, irrigating seedlings with warm water (surface water) can increase the stem thickness, leaf area, root coefficient, photosynthetic rate, dry matter per unit fresh weight, root-shoot ratio, and strong seedling index of seedlings [16]. Therefore, clarifying the effect of surface water and

underground water on cotton growth and yield by applying different irrigation regime is of paramount importance for improving crop productivity under limited water supply.

Xinjiang Uygur Autonomous Region is one of the most water-scarce states in China. According to the Statistics Bureau of Xinjiang Uygur Autonomous Region, the water production per unit area of Xinjiang Uygur Autonomous Region is 51 mm, which is the second highest rank in the country [19]. The large number of glaciers in Xinjiang Uygur Autonomous Region and the small unit area are important issues of water resources in Xinjiang Uygur Autonomous Region. Snowmelt water from glaciers accounts for more than 25% of total surface water [20]. However, according to China’s statistics, Xinjiang's total cotton output in 2017 was 4.082 million tons, accounting for 74.4% of the country's total production [20]. While on the other side, agriculture in Xinjiang is totally dependent on irrigation, according to 2016 Xinjiang Water Resource Bulletin, agriculture consumes 94.3% of the available total water resources.

In the present-day context, lot of emphasis is being given on improvements in irrigation practices to increase crop production and to sustain the productivity levels. In this study, two field experiments were carried out in calcareous soils. We hypothesize that surface water drip irrigation outcompetes underground water irrigation in increasing cotton yield, dry matter accumulation and NPK uptake. Therefore, the objectives of our study were to (i) compare the effects of different irrigation schemes on cotton growth and yield. (ii) Clarify the response of cotton growth to surface and underground water addition, (iii) finally put forward an appropriate irrigation strategy to increase cotton yield in calcareous soil.

2. Materials and Methods

2.1 Experimental site

The study area is located in the Mosuowan reclamation region (44° 03′ N, 86° 05′ E), which is located on the northern slope of Tianshan Mountain in Xinjiang and is surrounded by the Gurbantunggut Desert. According to WRB soil taxonomy, the tested soil is classified as Calcisol Fluvisols. The study area has a typical continental climate with a mean annual precipitation of 115 mm, and rainfall largely occurs from April to July. The mean annual potential evapotranspiration is approximately 2,000 mm. The physicochemical characteristics of given soil is listed in (Table 1).

Table 1. Selected physical and chemical properties of the tested soils.

**Soil properties**	**0-20cm**
pH	8.83±0.03
OM (g kg^{-1})	13.7±0.23
Total-N (g kg^{-1})	1.20±0.05
Available-P (mg kg^{-1})	17.5±3.52
Total-P (g kg^{-1})	1.08±0.12
Total-K (mg kg^{-1})	21.0±2.07
Available-K (mg kg^{-1})	241.4±0.00

Data were presented as the mean ± standard error (SE), n=3 at a significance level of $p < 0.05$.
a. pH was determined at soil to milli-Q water ratio of 1:5 w/v using pH meter.
b. Organic matter was measured by potassium dichromate volumetric method (Shaw, 1959) [37].
c. Total N was measured by the semimicro-Kjeldahl method (Bao 2000).
d. Total P was measured by the perchloric acid digestion method (Bao 2000).
e. Total and available K was measured by the flame photometry method (Bao 2000).

2.2 Experimental Design

The first experiment (different mixing ratio) was a randomized complete block design (RCBD) field experiment conducted on April 12, 2019 with five treatments including U (underground water); S (surface water); U:S= 1:1; U:S= 1:2; U:S= 1:3, respectively. Each treatment was replicated three times while the area of each block was 55.2 m^2. For the mixing ratio experiment we dig several whole and cover it with plastic, after that we supply surface and

underground water into the whole with a constant water ratio by using water ratio measurement instrument. After supplying water we mix the nutrient with the water and supply it to cotton field through drip irrigation. The schematic of our experimental design are presented in supplementary figure 1. The supply of water and nutrients for each irrigation has listed in (Table 2).

Insert "Supplementary figure 1 and (Table 2)"

Table 2. The amount of nutrients (fertilizer) and water during the period of cotton irrigation (mixing ratio irrigation 2019).

**Irrigation time**	**Irrigation water ($m^3.hm^{-2}$)**	**Nitrogen ($Kg.hm^{-2}$)**	**Phosphorus ($Kg.hm^{-2}$)**	**Potassium ($Kg.hm^{-2}$)**
12.6	450	30	15	15
20.6	450	75	75	75
30.6	375	105	105	105
10.7	375	105	105	105
20.7	300	75	150	150
31.7	300	30	180	180
10.8	225	75	75	75
24.8	225	45	45	45

The second experiment (round experiment) was a field randomized complete block design (RCBD), comprised of eight treatments including, 1:3 (T_1), 2:2 (T_2), 3:1 (T_3), 1:3 {S:U (T_4)}, 2:2 {S:U (T_5)}, 3:1 {S:U (T_6)}, 4:0 (T_7) and 0:4 (T_8). The treatment ratios represents the supply of surface and underground water in different stages of cotton growth i.e., seedling stage, growth stage, boll stage and boll opening stage. In round water irrigation we supply the constant ration of both surface water and underground water directly to the cotton field at various growth stages without mixing it. For maintaining the required ratios we first supply the specific ratio of surface water and then we supply the specific ratio of underground water by using a water ratio measurement meter. Further details about irrigation is given in (Table 3). Nutrients (fertilizer) and water were supplied through drip irrigation. The supply of water and nutrients for each irrigation has listed in (Table 4).

Insert "(Table 3 and 4)"

Table 3. Supply of surface and underground water at different growth stage of cotton (round irrigation 2020)

Treatments	Seedling stage	Growth stage	Boll stage	Boll opening stage
T1 U:S (1:3)	[1]U	S	S	S
T2 U:S (2:2)	U	U	S	S
T3 U:S (3:1)	U	U	U	S
T4 S:U (3:1)	[2]S	S	S	U
T5 S:U (2:2)	S	S	U	U
T6 S:U (1:3)	S	U	U	U
T7 U:S (4:0)	U	U	U	U
T8 U:S (0:4)	S	S	S	S

[1] U represents the supply of underground water.
[2] S represents the supply of surface water.

Table 4. The supply of water and nutrients (fertilizer) for each irrigation (round irrigation 2020).

Irrigation order	Water ($m^3/667m^2$)	Nitrogen ($Kg/667m^2$)	Phosphorus ($Kg/667m^2$)	Potassium ($Kg/667m^2$)
1	30	2	1	1
2	30	5	5	5
3	25	7	7	7
4	25	7	7	7
5	20	5	10	10
6	20	2	12	12
7	15	5	-	-
8	15	3	-	-

2.3 Soil and Plants sampling

Soil samples were collected at depths of 0-20 cm and 20-40 cm from each block after 3-5 days of irrigation. Samples were air dried, sieved through 1mm and 0.15 mm for nutrients determination. The pH was determined at soil to milli-Q water ratio of 1:5 w/v using pH meter. The organic matter was determined by potassium dichromate volumetric method. Nitrogen was determined by semi micro-Kjeldahl method [37]. Phosphorus was measured by the per chloric

acid digestion method [38]. Potassium was determined by flame photometry [37]. Soil available Nitrogen, soil available phosphorus, soil available potassium and soil organic matter were determined in soil samples after growth stage, boll stage and boll opening stage.

Plant samples were randomly collected from each block at interval of 3-5 days after each irrigation. Plant samples were divided into the following parts (leaves, stems, roots and fruits), washed with tap water and then dried in oven at 105˚C for 30 minutes and then at 75˚C for 3 days. The plant samples were then weighed with balance and the dry matter data were calculated after growth stage, boll stage and boll opening stage, following the method of [39] (Figure 1).

Insert "(Figure 1)"

2.4 Statistical analysis

Data were analyzed using the SPSS 25.5 statistical program (SPSS Inc., Chicago, IL, USA) with ANOVA at a significance level of $p < 0.05$. A Duncan multiple range test was carried out to test the significant differences between different treatments. GraphPad Prism 12.0 software (GraphPad Software, Inc., San Diego, CA, USA) was used for data processing and images making. All results in figures and tables were presented as mean ± standard deviation (SD) of three replicates, and a significance level of $p < 0.05$ was used for all analysis.

3. Results

3.1 Effects of different mixing ratio irrigation method on the dry matter accumulation

The average dry matter accumulation in the different parts of the cotton plant fluctuated greatly with different irrigation treatments and increased sharply after each irrigation time (Figure. 2). For instance, the significant maximum dry weights (DW) were recorded in S treatment followed by the U: S= 1:3 treatment. However, the lowest dry matter accumulation

was recorded in the U treatment. The concentration of leaves dry matter after 8th irrigation increased from 32.8 ± 0.15 g (U), to 74.1 ± 0.27g (S), 43.5 ± 0.18g (U:S= 1:1), 51.1 ± 0.21g (U:S= 1:2) and 62.9 ± 0.37g (U:S= 1:3), with an average increase rate of 131.2%, 34.4%, 59.3%, and 93.7%, respectively ($p < 0.05$, Figure 2a). Compared with underground water the tremendous average increase rate of 131.2% and 93.7% were noted in surface water and different mixing ratios. Similarly, stem dry matter of five applied treatments as affected by eight irrigation regimes are presented in Figure 2b. The concentration of stem dry matter after 8th irrigation increased from 48.5 g (U), to 122.2 g (S) and 101.6 g (U:S= 1:3), with an average increase rate of 151.5 % and 109.5%, (Figure 2b). Moreover, compared with underground water irrigation treatment (24.6 g), a significant highest root dry matter was recorded with surface water treatment (51.74 g) and U: S= 1:3 treatment (46.96 g) ($p < 0.05$, Figure 2c). Similarly, compared with surface water, underground water significantly suppressed roots dry matter accumulation and follows the sequence of S> U: S= 1:3> U: S= 1:2> U: S= 1:1>U. The highest fruits dry matter accumulation was recorded in the treatment of surface water (83.43 ± 0.34 g) compared with all other treatments ($p < 0.05$, Figure 2d).

Insert "(Figure 2)"

3.2 Effects of round irrigation method on dry matter accumulation

Round irrigation method also significantly affected dry matter accumulation in the different parts of the plant at various stages of growth ($p < 0.05$, Figure. 3). The biomass of yield-related organs showed a trend of gradual increase with the progression of the growth period, and the most intense change was at the beginning of the boll development stage. Maximum dry weights (DW) were achieved at boll opening stage and minimum at growth stage. The DW of

leaves, stem, roots and fruits continued to increase initially and then declined and stayed stable comparatively because of leaf senescence and termination of reproductive development at final stages (Figure 3a-d). The highest leaves dry matter of 14.4g (growth stage), 88.8g (boll stage) and 100.7g (boll opening stage) were observed in T8 treatment compared with the lowest, 8.9g (growth stage), 42.9g (boll stage) and 78.7g (boll opening stage) of T7 treatment. Results of Two-way ANOVAs reveals a significant main and interactive effect on various stage of leaves dry matter treated with surface water ($p < 0.05$, Figure. 3a). Compared with T7 treatment an average increase rate of 50.6 %, 100.9 % and 93.3 %, at growth, boll and boll opening stage respectively in stem dry matter were noted in T8 treatment. Likewise, a significant main and interactive effect were observed at various stages of stem dry matter ($p < 0.05$, Figure. 3b). Also, it was evident from the results that the highest root dry matter recorded in growth, boll and boll opening stage were 6.46g, 38.68g and 51.63g with T8 treatment, compared with the lowest values of 4.60g, 12.24g and 23.57g recorded in T7. A significant main and interactive effect were observed at boll and boll opening stages of roots dry matter ($p < 0.05$, Figure. 3c). Fruits dry matter were produced in boll and boll opening stages and was substantially higher in T8 treatment compared with other applied treatments throughout the whole experiment. A significant main interaction effect on boll and boll opening stage while a significant interactive effect on boll stage were observed for fruits dry matter ($p < 0.05$, Figure. 3d). Our result showed that the surface water irrigation along with mixing ratio shows promising effect on average dry matter accumulation in cotton plant.

Insert "(Figure 3)"

3.3 Effects of different mixing ratio and round irrigation on soil available nutrients

Soil available nitrogen at a depth of 0–20 and 20–40 cm fluctuated greatly during the whole cotton growth period, and increased sharply after each irrigation time (Figure. 4a, b). The concentration of N at 0–20 cm after 8th irrigation increased from 72.3 ± 0.15 mg/kg (U), to 101.3 ± 0.27 mg/kg (S), 77.1 ± 0.18 mg/kg (U:S=1:1), 82.1 ± 0.21 mg/kg (U:S=1:2) and 99.3 ± 0.37 mg/kg (U:S=1:3), with an average increase rate of 40.1%, 6.6%, 13.5%, and 29.5%, respectively ($p < 0.05$, Figure 4a). Compared with U treatment an average increase in N concentration at 20–40 cm after 8th irrigation in S treatment were 37.4 %, 7.1%, 20.0%, and 21.9% ($p < 0.05$, Figure 4b). Trends of soil available nitrogen follows the order of S> U:S=1:3> U:S=1:2> U:S=1:1>U. Moreover, the accumulation of P in different soil depths 0–20 and 20–40 cm was presented in (Figure. 4c, d). The concentration of P at 0–20 cm after 8th irrigation increased by 76.9%, and 33.8% %, respectively in S and U:S=1:3 treatments compared with U treatment ($p < 0.05$, Figure 4c). Whilst at 20-40 cm, the amount of P in surface water application significantly increased from 12.6 ± 0.15 mg/kg (U), to 28.4 ± 0.27 mg/kg (S), 13.4 ± 0.18 mg/kg (U:S=1:1), 13.3 ± 0.21 mg/kg (U:S=1:2) and 22.6 ± 0.37 mg/kg U:S=1:3 ($p < 0.05$, Figure 4d). The status of available potassium in soil at 0–20 and 20–40 cm remains parallel throughout the experiment (Figure 5a, b). Compared with other treatments the surface water treatment shows maximum concentration of potash, however the difference between different applied treatments were negligible. Soil organic matter was significantly affected by different irrigation treatments (Fig. 5c, d). However, at the start of the experiment the S treatment followed by U: S=1:3 treatment shows potential promising effect at both 0–20 and 20–40 cm, while the difference between other treatments were negligible.

Furthermore, the concentration of soil available nutrients at a depth of 0-20cm and 20-40

cm in round irrigation method significantly affected by different applied treatments. The concentration of N recorded at 0–20 cm at different growth stages were 83.3±2.8 (growth stage), 79.01±1.84 (boll stage), 96.16±3.83 (boll opening stage) in T8, while in T7 the concentration of N was 36.1±5.9 (growth), 54.51±2.81 (boll), and 53.9±3.83 (boll opening) ($p < 0.05$, Table 5). The soil available P content significantly increased in the initial and final stages with T8 treatment throughout the experiment ($p < 0.05$, Table 6). While, the status of available potassium in soil remains parallel throughout the experiment, the difference between different applied treatments were negligible ($p < 0.05$, Table 7). However, the maximum concentration of potash was determined in T8 treatment. Soil organic matter was significantly affected by different irrigation treatments ($p < 0.05$, Table 8). However, at the start of the experiment the surface water irrigation treatment shows promising effect at both 0–20 and 20–40 cm, while the difference between other treatments were negligible.

Insert "(Figure 4 and Figure 5)"

Insert "(Table 5, Table 6, Table 7 and Table 8)"

Table 5. Effect round irrigation method on soil available nitrogen (mg/kg). Data were presented as the mean ± standard deviation (SD) of three replicates at a significance level of $p < 0.05$ (based on ANOVA).

Soil depth	Treatments	Growth stage	Boll stage	Boll opening stage
0-20 cm	1	77.8±3.8a	77.18±1.84a	78.4±3.83b
	2	59.4±4.6b	75.34±1.84a	75.34±4.86b
	3	37.4±3.8d	67.38±2.81b	60.03±3.83cd
	4	44.7±3.8c	74.11±4.62a	63.09±4.62c
	5	39.8±2.8d	67.38±3.83b	61.25±2.81cd
	6	37.1±2.3d	67.38±1.06b	57.58±3.83cd
	7	36.1±5.9d	54.51±2.81c	53.9±3.83d
	8	83.3±2.8a	79.01±1.84a	96.16±3.83a
20-40 cm	1	55.7±3.8a	50.84±2.81a	52.37±1.3ab
	2	45.9±1.8b	34.91±1.84b	53.9±2.81a
	3	28.2±1.1c	27.56±4.86bc	44.1±1.84abc
	4	29.7±2.3c	33.69±3.83b	47.78±4.86ab
	5	28.2±2.8c	29.4±5.51bc	47.78±1.84ab
	6	22.4±8cd	24.5±2.81c	41.65±3.83bc
	7	17.2±3.8d	23.89±4.86c	33.69±7.65c
	8	60.6±4.9a	52.68±4.62a	47.16±12.23ab

Table 6. Effect round irrigation method on soil available phosphorus (mg/kg). Data were presented as the mean ± standard deviation (SD) of three replicates at a significance level of $p < 0.05$ (based on ANOVA).

Soil depth	Treatments	Growth stage	Boll stage	Boll opening stage
0-20 cm	1	30.89±0.27ab	30.23±0.45b	28.36±0.22b
	2	29.98±0.58ab	28.33±0.44c	28.01±0.29b
	3	22.56±1.68c	23.14±0.25f	20.13±0.21e
	4	25.72±6.69bc	27.52±0.21d	23.75±0.12c
	5	22.91±4.52c	25.3±0.49e	22.36±0.93d
	6	20.51±4.61c	19.47±0.55g	19.18±0.3f
	7	20.23±4.06c	19.11±0.37g	18.18±0.19g
	8	34.44±1.67a	31.18±0.2a	31.52±0.37a
20-40 cm	1	19.33±0.69a	18.64±0.07ab	16.69±0.36b
	2	17.84±5.36a	18.03±1.65ab	14.95±0.3c
	3	12.13±0.62bc	17.04±0.3bc	13.44±0.29d
	4	16.79±0.63ab	17.93±0.3ab	14.52±0.28c
	5	17.88±2.49a	17.18±1.95bc	14.47±0.53c
	6	11.48±2.32c	15.93±0.63c	11.12±0.42e
	7	9.72±4.77c	12.66±0.29d	11.17±0.62e
	8	19.69±0.81a	19.11±0.39a	18.15±0.22a

Table 7. Effect round irrigation method on soil available potassium (mg/kg). Data were presented as the mean ± standard deviation (SD) of three replicates at a significance level of $p < 0.05$ (based on ANOVA).

Soil depth	Treatments	Growth stage	Boll stage	Boll opening stage
0-20 cm	1	471.42±2.4b	481.32±2.51a	451.62±3.07b
	2	457.99±2.58c	473.16±5.52b	433.71±2.05c
	3	418.3±3.07f	464.16±2.84c	418.35±3.34e
	4	442.24±2.83d	470.73±2.56b	429.47±1.43c
	5	431.85±1.84e	469.95±1.25b	423.96±4d
	6	400.64±2.07g	462±2.17c	408.89±3.04f
	7	368.54±5.57h	429.11±2.35d	373.68±1.54g
	8	496.89±2.7a	483.81±2.73a	473.05±3.19a
20-40 cm	1	401.43±2.58b	376.18±8.94a	406.25±1.89b
	2	368.45±1.46c	367.14±0.1b	386.44±3.22c
	3	346.49±2.47f	355.5±4.22c	361.91±3.15f
	4	362.91±2.31d	365.74±1.57b	377.18±2.32d
	5	355.72±3.57e	360.36±3.52bc	370.62±3.39e
	6	333.71±2.68g	332.15±4.61d	346.2±2.94g
	7	322.19±3.16h	324.94±3.11d	324.11±1.36h
	8	434.53±3.14a	383.16±3.84a	428.69±2.04a

Table 8. Effect round irrigation method on soil organic matter (g/kg). Data were presented as the mean ± standard deviation (SD) of three replicates at a significance level of $p < 0.05$ (based on ANOVA).

Soil depth	Treatments	Growth stage	Boll stage	Boll opening stage
0-20 cm	1	13.3371±0.33569b	12.4866±0.68639ab	13.1195±0.11468a
	2	13.0145±0.58669bc	11.0622±0.35745abc	12.0848±0.09785b
	3	11.7308±0.36465ef	10.5849±0.31272c	11.2333±0.95552bc
	4	12.5637±0.32678cd	10.9999±0.64795abc	11.5946±0.31635bc
	5	12.2023±0.4165de	10.7173±0.41908bc	11.3057±0.30968bc
	6	11.4874±0.43846ef	10.252±0.71663c	11.1814±0.83826bc
	7	11.3291±0.37229f	10.2181±0.24148c	10.8893±0.12844c
	8	14.4407±0.2702a	12.6024±2.42886a	13.427±0.36673a
20-40 cm	1	10.6436±0.28389a	9.868±0.28809a	10.6518±0.2795a
	2	10.4489±0.13549ab	9.687±0.22901a	10.4804±0.32128a
	3	9.9319±0.71469ab	9.3676±0.16334a	9.7692±0.25222b
	4	10.1805±0.09637ab	9.5965±0.53775a	9.9897±0.22095b
	5	10.1261±0.41629ab	9.4632±0.52158a	9.9327±0.27535b
	6	9.458±0.22105bc	9.1858±0.38905a	9.6588±0.11612b
	7	8.6652±0.77305c	9.1339±0.53729a	9.6424±0.21155b
	8	10.7595±0.99849a	9.911±0.37617a	10.7889±0.2811a

3.4 Effects of different irrigation methods on cotton yield

The effect of different mixing ratio irrigation on cotton yield is presented in Figure 6a. Cotton yield responded significantly to different water irrigation modes. For instance, the maximum cotton yield 6571 kg/hm^2 was observed in (S) treatment compared with the treatment (U) (5492 kg/hm^2), U: S= 1:1 treatment (5502 kg/hm^2), U: S= 1:2 treatment (5873 kg/hm^2) and U: S= 1:3 treatment (6111 kg/hm^2). Likewise, round irrigation potentially effect cotton yield under different applied treatments (Figure 6b). For example, the increasing trend follow the order of T8 > T1 > T4 > T2 > T5 > T3 > T6> T7, which shows that surface water irrigation can effectively guarantee cotton production. The highest cotton yield was observed in T8 treatment in which surface water was supplied through all stages of cotton growth, followed by treatment T1

and T4 (Figure 6b). However, the lowest yield was recorded in treatment T7 in which underground water was supplied.

Insert "(Figure 6)"

4. Discussion

Xinjiang region is one of the most important cotton producers with the plantation area of about 1.8 × 106 ha, accounting for 54% of China's total cotton planting area [20]. Approximately, about 451 × 104 tons of cotton were produced in 2014, accounting for 73% of China's total cotton production [18]. Meanwhile, surface water evaporation caused by high temperatures results in a severe water shortage in southern Xinjiang leading to soil salinization, a lowered survival rate for crops, and slow development of local agriculture [21]. Similarly, increased usage of underground water for irrigation exacerbates the soil salinization problems, which significantly reduce crop yield [10, 11]. In this study, the application of surface water in both mixing ratio and round irrigation methods, significantly promotes dry matter accumulation (Fig. 2-3), NPK uptake (Fig. 4,5 and Table 5-8) and cotton yield (Fig. 6), compared with all other applied treatments. Our obtained results are in line with the findings of previous published literature [34, 35, 36]. The superiority of surface water over underground water in calcareous soil may likely be due to the following reasons; in general surface water possess high temperature while the underground water temperature is quite low. Previous study has shown that well water irrigation with low temperature potentially inhibits the growth and development of jujube [22]. Likewise, several published literatures have shown that underground water irrigation (low temperature water) affect the growth, yield, dry matter accumulation and active developmental stages of grains plants such as peanuts, cucumber, and tomato [23, 24, 25, 26]. For instance, a

study conducted by Meng et al. (2016), noted that underground water irrigation significantly affects the growth and development of cotton plant. Similar results with the application of underground water irrigation is also obtained in this study. Furthermore, Deng et al., [27], also pointed out that underground water irrigation along with their low temperature properties significantly retarded the growth of vegetables and their photosynthetic developments. Consequently, the excessive usage of underground water irrigation results in the accumulation of toxic substances in soil which alternatively leads to reduction in plants and grains yields [28, 29]. The accumulation of salt can directly decrease soil nutrient efficiency by inhibiting microbial mineralization activity in soil [30]. Additionally, salinity can also indirectly affect soil nutrient cycling and efficiency by destroying soil physical structure [31, 32, 33]. On the flip side, the study of Zhang et al., 2002 [34] showed that alternate irrigation with surface fresh water can reduce soil salt content and increase cotton production which are in line with our findings. In this study surface water irrigation along with different mixing ratios irrigation also shows promising effects on cotton yield and dry matter accumulation when compared with underground water treatment alone, this is possibly due to when the underground water and surface water were mixed together, the temperature and salt content were changed. For example, a study conducted by Tao et al. 2014 [35], showed that mixed irrigation mode of brackish and fresh water with a salinity of 1.6g/L could achieve higher crop yield with better quality. Consistently, a study carried out by Wang et al. 2010 [36], showed that well and canal mixed irrigation could keep the salt balance of root soil even in relatively dry years, while well irrigation alone results in salt accumulation in roots of winter wheat and decreased the yield up to 20% -30%. All these findings suggest that under-ground water irrigation possess negative effects on plant yield and growth whilst surface water irrigation and different mixing ratios irrigation significantly promote

cotton yield, NPK uptake and dry matter accumulation.

5. Conclusions

It can be concluded that the application of surface water along with their different mixing ratios irrigation outcompete underground water irrigation in both mixing ratio and round irrigations methods. A significant highest dry mater accumulation, nutrients uptake and cotton yield at various stages i.e., growth stage, boll stage, and boll opening stage were always noted surface water applied treatments compared with underground water treatment. Overall, our findings provide meaningful information to current irrigation practice in increasing cotton growth and yield. Therefore taken into account the scarcity of water resource surface water irrigation application is recommended as an effective irrigation strategy in Xinjiang calcareous soil for better cotton yield and nutrient uptakes.

Disclosure statement

The authors declare that they have no conflict of interest.

References

1. UN-Water. The United Nations World Water Development Report 4: Managing Water under Uncertainty and Risk; United Nations World Water Assessment Programme, UNESCO: Paris, France, 2012.
2. Gu Z, Qi Z, Ma L, Gui D, Xu J, Fang Q, Yuan S, Feng G. Development of an irrigation scheduling software based on model predicted crop water stress. Comput. Electron. Agric. 2017; 143, 208–221.
3. Feike T, Ling YK, Mamitimin Y, Nan H, Lin L, Abdusalih N, Xiao H, Doloschutz R. Determinants of cotton farmers’ irrigation water management in arid northwestern china. Agric. Water Manag. 2017; 187, 1–10.
4. Wei Qu, Yanmei Tan, Zhentao Li, Eefje Aarnoudse, Qin Tu. Agricultural Water Use Efficiency—A Case Study of Inland-River Basins in Northwest China. Sustainability. 2020; 12, 10192.

5. Meng B, Liu JL, Bao K, Sun B.Water fluxes of Nenjiang river basin with ecological network analysis: Conflict and coor-dination between agricultural development and wetland restoration. J. Clean. Prod. 2019; 213, 933–943.
6. Liu Y, Jiang H, Li C, Huang H, Pan Z, Chai C. Analysis of irrigation water requirement and irrigation requirement index for cotton of Hebei province. Nongye Gongcheng Xuebao/Trans. Chin. Soc. Agric. Eng. 2013; 29, 98–104.
7. Li Y, Gao X, Tenuta M, Gui D, Li X, Xue W, Zeng F. Enhanced efficiency nitrogen fertilizers were not effective in reducing N2O emissions from a drip-irrigated cotton field in arid region of Northwestern China. Sci. Total Environ. 2020; 748, 141543.
8. FAOSTAT. 2021. Available online: http://www.fao.org/faostat/en/#data/QCL. (accessed on 25 July 2021).
9. Ma C, Mamat S, Yao J, Isak G. Spatio-temporal changes of cotton production in China from 1950 to 2015. Acta Geogr. Sin. 2020; 75, 1699–1710.
10. Sorensen RB, Lamb MC, Butts CL. Crop rotation, irrigation system, and irrigation rate on cotton yield in southwestern Georgia. Crop. Forage Turfgrass Mgmt. 2020; 6, e20053.
11. Mahmoodi-Eshkaftaki M, Rafiee MR. Optimization of irrigation management: a multi-objective approach based on crop yield, growth, evapotranspiration, water use efficiency and soil salinity. Journal of Cleaner Production. 2020; 252, 119901.
12. Evett SR, Baumhardt RL, Howell TA, Ibragimov NM, Hunsaker DJ. Cotton. Crop Yield Response to Water; FAO ir-rigation and drainage paper. No. 66; FAO: Rome, Italy. 2012; pp. 152–161.
13. Grismer ME. Regional cotton lint yield, ETc, and water value in Arizona and California. Agric. Water Manag. 2002; 54, 227–242.
14. Sekhon KS, Kaur A, Thaman S, Sidhu A S, Garg N, Choudhary O P, Buttar G S, Chawla N. Irrigation water quality and mulching effects on tuber yield and soil properties in potato (*Solanum tuberosum* L.) under semi-arid conditions of Indian Punjab. Field Crops Research. 2020; 247, 107544.
15. Meng A. Effect of drip irrigation temperature on grey desert soil environment and cotton growth. Urumqi: Xinjiang Agricultural University. 2016; 39-40.
16. Kong Q. Analysis on the key factor of inhibiting cotton production development and discussion on its strategies. Xinjiang Agric. Sci. 2010; 47, 3–5 (in Chinese with English abstract).
17. Kang Yaohu, Wang Ruoshui, Wan Shuqin, Hu Wei, Jiang Shufang, Liu Shiping. Effects of different water levels on cotton growth and water use through drip irrigation in an arid region with saline ground water of Northwest China. Agric. Water Manag. 2013; 109, 117– 126.
18. Chen Z, Niua Y, Zhaoa R, Hana C, Hanb H, Luoa H. The combination of limited irrigation and high plant density optimizes canopy structure and improves the water use efficiency of cotton. Agric. Water Manag. 2019; 218, 139–148.
19. Wang J, Zhao S, Tan X, Liang Y, Gong Z, Ai X, Guo J, Maimaiti M, Li X, Zheng J. Production situation and cotton seed industry development report in Xinjiang cotton planting region in 2018. Cotton Sci. 2019; 41, 9–14.

20. Tian JS, Zhang XY, Yang YL, Yang CX, Xu SZ, Zuo WQ, Zhang WF, Dong HY, Jiu X L, Yu Y C, Zhao Z. How to reduce cotton fiber damage in the Xinjiang China. Industrial Crops and Products. 2017; 109, 803-811.
21. Fang S, Tu W, Mu L, Sun Z, Yang Y. Saline alkali water desalination project in southern Xinjiang of China: a review of desalination planning, desalination schemes and economic analysis. Renewable and Sustainable Energy Reviews. 2019; 113, 109268.
22. Zhang RW, Tian JC, Ma JM. The effect of different irrigation water temperatures on the growth and photosynthesis of leaf lettuce . China Rural Water and Hydropower. 2017; 4, 1 - 2.
23. Bai YY, Wang HD, Li M. et al. Effect on different irrigation water temperatures on tomato seedlings growth in greenhouse. Water Saving Irrigation. 2012; 11, 16 - 17.
24. Shi PX, Liu YR, Zhang XJ. et al. Effects of irrigation with low temperature water on soil enzyme activity and soil nutrient of peanut rhizosphere. Chinese Journal of Oil Crop Sciences. 2016; 38, 811 - 816.
25. Kuang W, Gao X, Gui D, Tenuta M, Flaten DN, Yin M, Zeng FJ. Effects of fertilizer and irrigation management on nitrous oxide emission from cotton fields in an extremely arid region of northwestern China. Field Crop Research. 2018; 229, 17-26.
26. Sun SJ, Cui SM, Song Y. et al. Effects of root zone temperature on growth and photosynthetic parameters of grafted cu-cumber. Agricultural Biotechnology. 2018; 7, 27 - 31.
27. Deng HL, Tian JC, Ouyang Z. Effect of Irrigation water temperature on soil temperature in vegetable root zone in greenhouse. Ningxia Engineering Technology. 2018; 17, 97 - 101.
28. Bumgaer NR, Scheerens JC, Mullen RW, et al. Root-zone temperature and nitrogen affect the yield and secondary me-tabolite concentration of fall and spring grown, high-density leaf lettuce. Journal of the Science of Food ＆ Agriculture. 2012; 92, 116 - 124.
29. Li LJ, Lu XC, Ma HY. et al. Comparative proteomic analysis reveals the roots response to low root-zone temperature in Malus baccata. Journal of Plant Research. 2018; 131, 865 - 878.
30. Rath KM, Rousk J. Salt effects on the soil microbial decomposer community and their role in organic carbon cycling: a review. Soil Biology Biochemistry. 2015; 81, 108-123.
31. Lakhdar A, Rabhi M, Ghnaya T, Montemurro F, Jedidi N, Abdelly C. Effectiveness of compost use in salt-affected soil. Journal of Hazardous Materials. 2009; 171, 29-37.
32. Zhang KF, Bosch-Serra AD, Boixadera J, Thompson AJ. Investigation of water dynamics and the effect of evapo-transpiration on grain yield of rainfed wheat and barley under a mediterranean environment: a modelling approach. Plos One. 2015; 10.
33. Zhang YY, Hua RX, Xia R. Impact analysis of climate change on water quantity and quality in the Huaihe River Basin. J. Nat. Resour. 2018; 1, 114–126.

34. Zheng J. Study on the utilization of brackish water under the condition of bar cover. Shandong Agricultural University. 2002;
35. Tao J, Tian J, Li J. Study on drip irrigation system of greenhouse pepper under different brackish water film. China Rural Water and Hydropower. 2014; 5, 68-72. (in Chinese).
36. Wang R, Kang Y, Wan S, Hu W, Liu S, Liu S. Salt distribution and the growth of cotton under different drip irrigation regimes in a saline area, Agricultural Water Management. 2011cvol; 100, no. 1, pp. 58–69.
37. Bao SD. Analysis of soil and agrochemistry. Third Edition. Beijing: China Agriculture Press. 2000; [in Chinese].
38. Olsen SR, Cole CV, Watanable FS, Dean L. Estimation of available phosphorus in soils by extraction with sodium bicarbonate. Washington, D.C. United States Department of Agriculture. 1954.
39. Makhdum MI, Pervez H, Ashraf M. Dry matter accumulation and partitioning in cotton (Gossypium hirsutum L.) as in-fluenced by potassium fertilization. Biol Fertil Soils. 2007; 43: 295–301.

Figure Click here to access/download;Figure;Figures.doc

Figure Captions

Figure 1: Schematic of dry matter accumulation calculation.

Figure 2. Effects of different mixing ratio irrigation methods on the accumulation dry matter: Figure (2a) represents leaves dry matter; (2b) represents stem dry matter; (2c) represent roots dry matter; and (2d) represent fruits dry matter, respectively. Data were presented as the mean ± standard deviation (SD) of three replicates at a significance level of $p < 0.05$ (based on ANOVA).

Figure 3. Effects of round irrigation methods on dry matter accumulation: Figure (3a) represents leaves dry matter; (2b) represents stem dry matter; (3c) represent roots dry matter; and (3d) represent fruits dry matter, respectively. Data were presented as the mean ± standard deviation (SD) of three replicates at a significance level of $p < 0.05$. The inserted P values are from two-way ANOVAs.

Figure 4. Effects of different mixing ratio irrigation on soil available Nitrogen (mg/kg) and soil available phosphorus (mg/kg): Figure (4a, b) represents soil available Nitrogen at depth of 0-20 cm and 20-40 cm; figure (4 c, d) represents soil available Phosphorus at depth of 0-20 cm and 20-40 cm. Data were presented as the mean ± standard deviation (SD) of three replicates at a significance level of $p < 0.05$ (based on ANOVA).

Figure 5. Effects of different mixing ratio irrigation on soil available Potassium (mg/kg) and soil organic matter (g/kg): Figure (5a, b) represents soil available potassium at depth of 0-20 cm and 20-40 cm; figure (5c, d) represents soil organic matter at depth 0-20 cm and 20-40 cm. Data were presented as the mean ± standard deviation (SD) of three replicates at a significance level of $p < 0.05$ (based on ANOVA).

Figure 6. Effects of different irrigation methods on cotton yield: figure (2019) represents Experiment I mixing ratio irrigation method; figure (2020) represents Experiment II Round irrigation method. Data were presented as the mean ± standard deviation (SD) of three replicates at a significance level of $p < 0.05$ (based on two-way ANOVA).

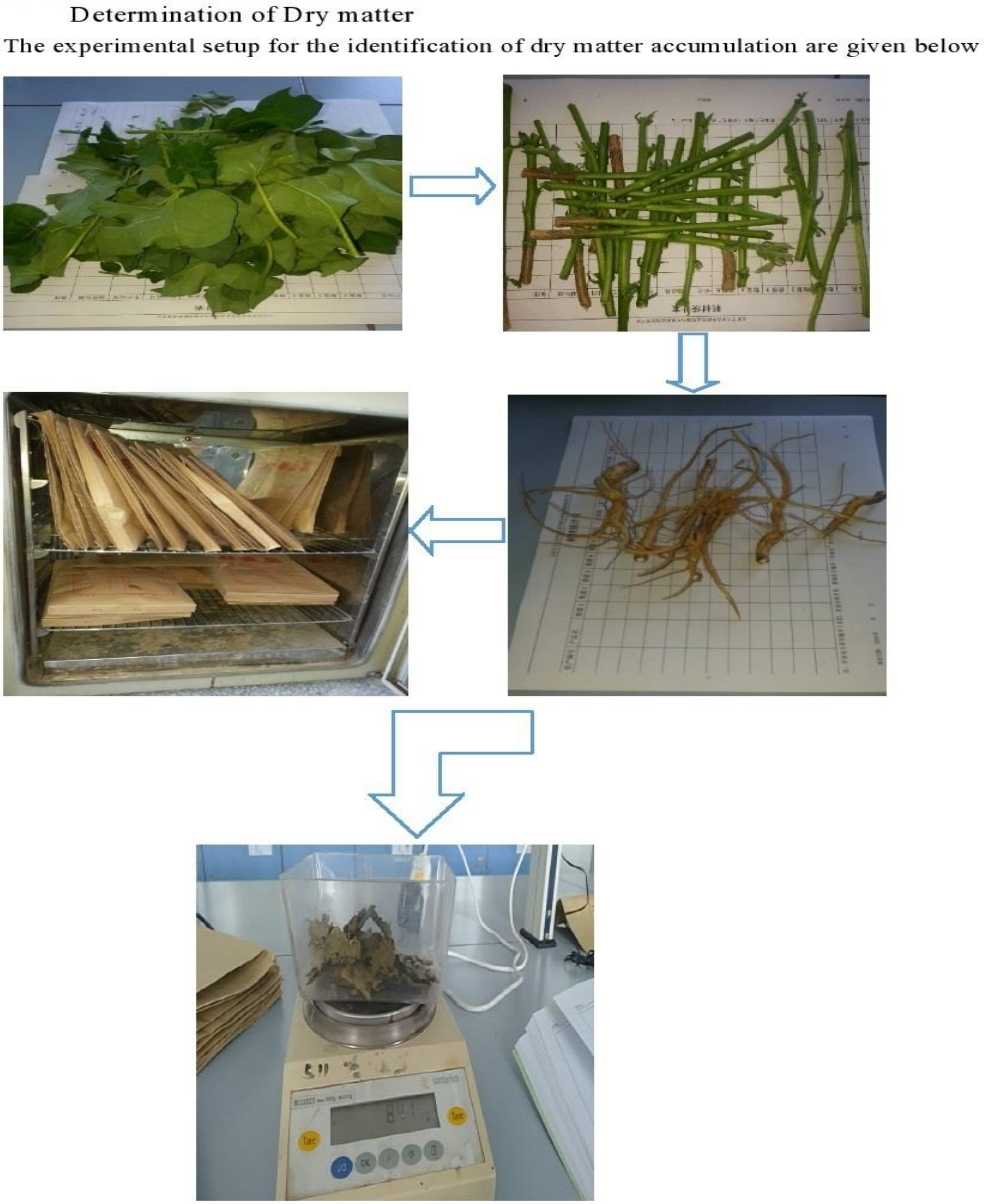

Figure 1. Schematic of dry matter accumulation calculation.

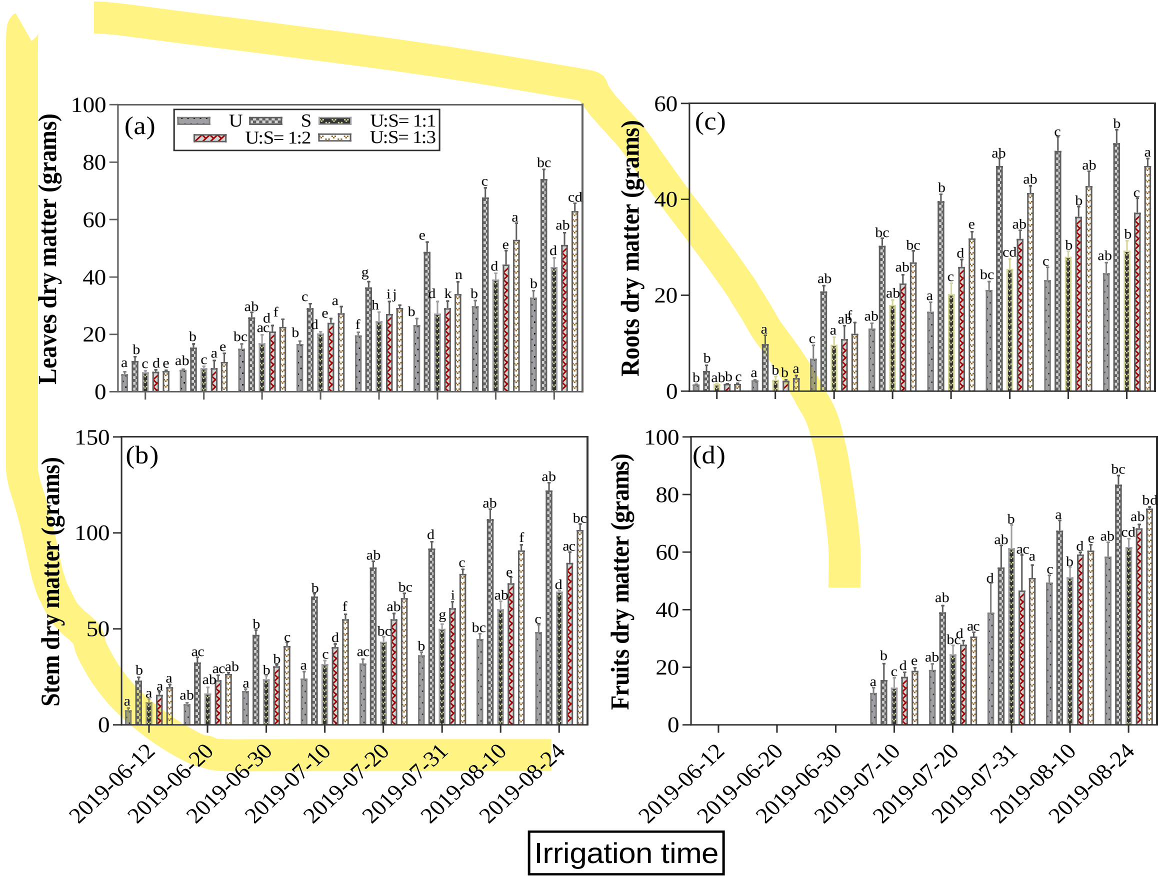

Figure 2. Effects of different mixing ratio irrigation methods on the accumulation dry matter: Figure (2a) represents leaves dry matter; (2b) represents stem dry matter; (2c) represent roots dry matter; and (2d) represent fruits dry matter, respectively. Data were presented as the mean ± standard deviation (SD) of three replicates at a significance level of $p < 0.05$ (based on ANOVA).

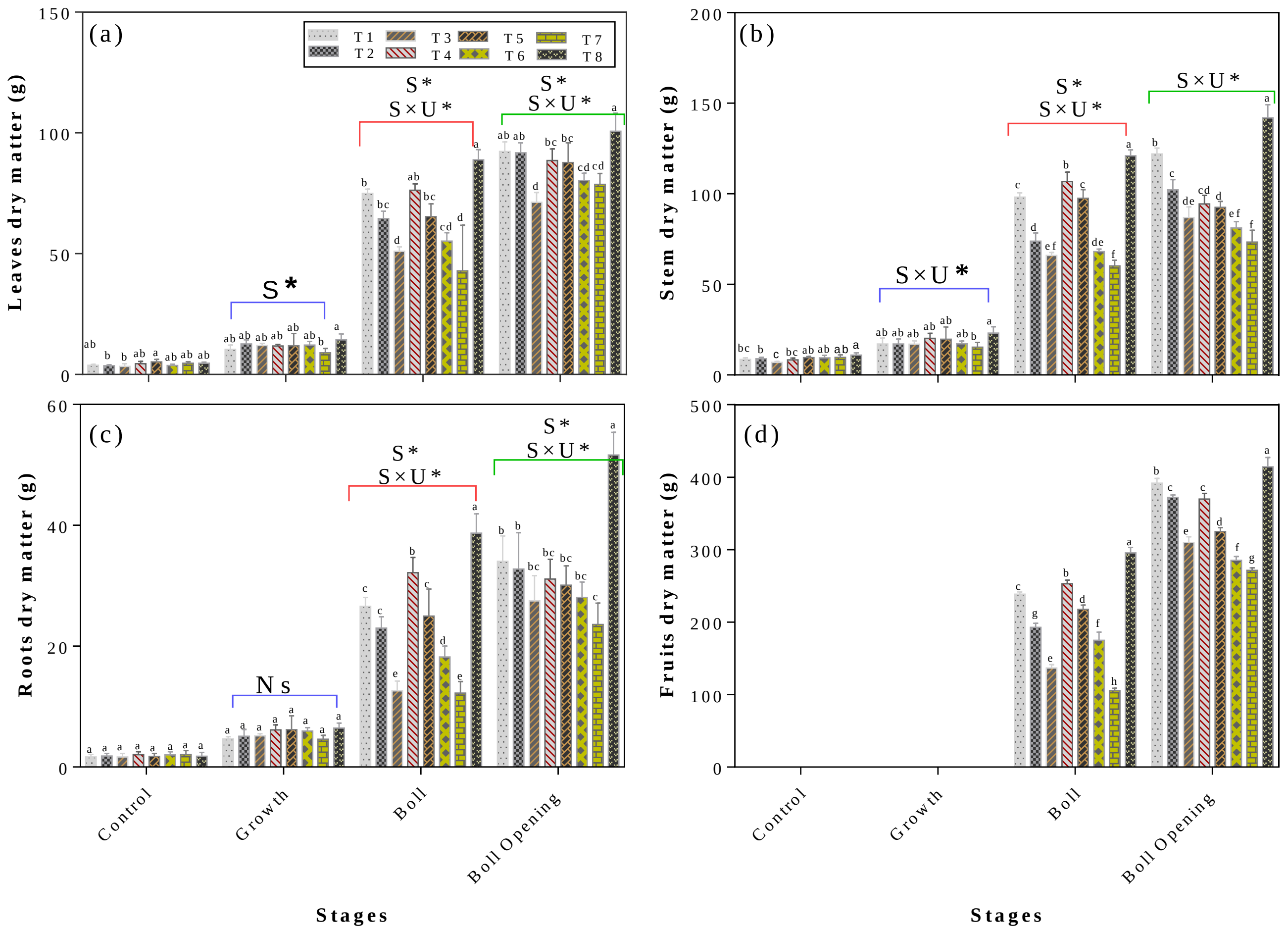

Figure 3. Effects of round irrigation methods on dry matter accumulation: Figure (3a) represents leaves dry matter; (2b) represents stem dry matter; (3c) represent roots dry matter; and (3d) represent fruits dry matter, respectively. Data were presented as the mean ± standard deviation (SD) of three replicates at a significance level of $p < 0.05$. The inserted P values are from two-way ANOVAs.

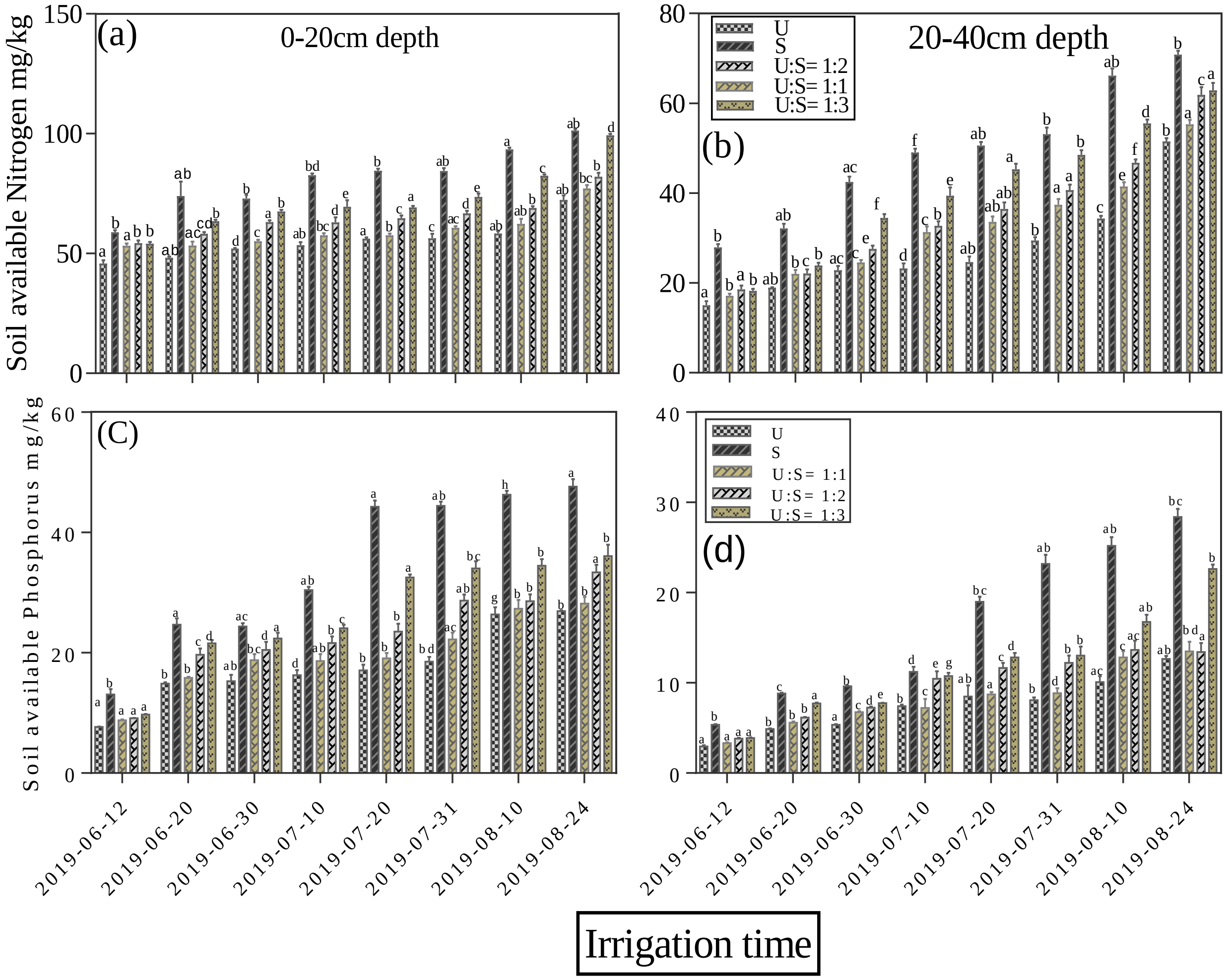

Figure 4. Effects of different mixing ratio irrigation on soil available Nitrogen (mg/kg) and soil available phosphorus (mg/kg): Figure (4a, b) represents soil available Nitrogen at depth of 0-20 cm and 20-40 cm; figure (4 c, d) represents soil available Phosphorus at depth of 0-20 cm and 20-40 cm. Data were presented as the mean ± standard deviation (SD) of three replicates at a significance level of $p < 0.05$ (based on ANOVA).

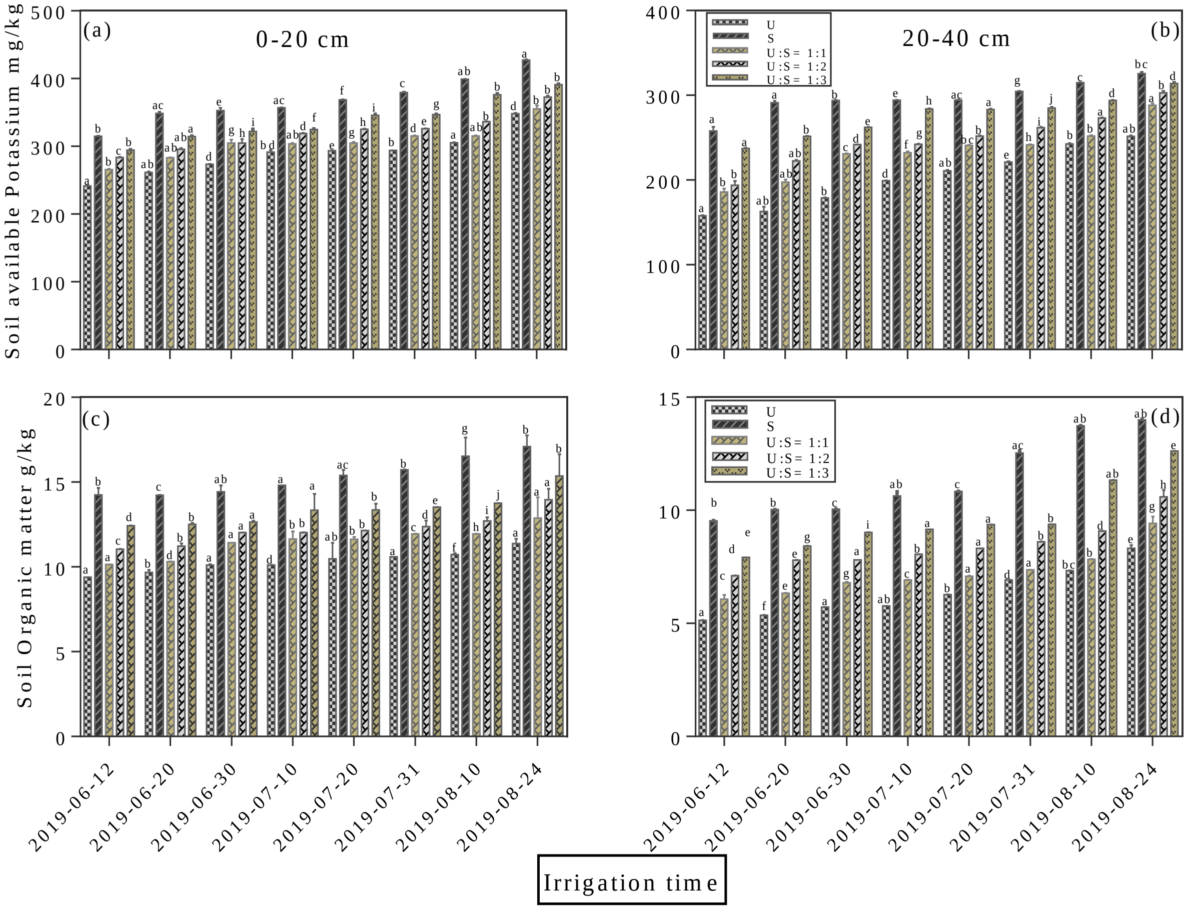

Figure 5. Effects of different mixing ratio irrigation on soil available Potassium (mg/kg) and soil organic matter (g/kg): Figure (5a, b) represents soil available potassium at depth of 0-20 cm and 20-40 cm; figure (5c, d) represents soil organic matter at depth 0-20 cm and 20-40 cm. Data were presented as the mean ± standard deviation (SD) of three replicates at a significance level of $p < 0.05$ (based on ANOVA).

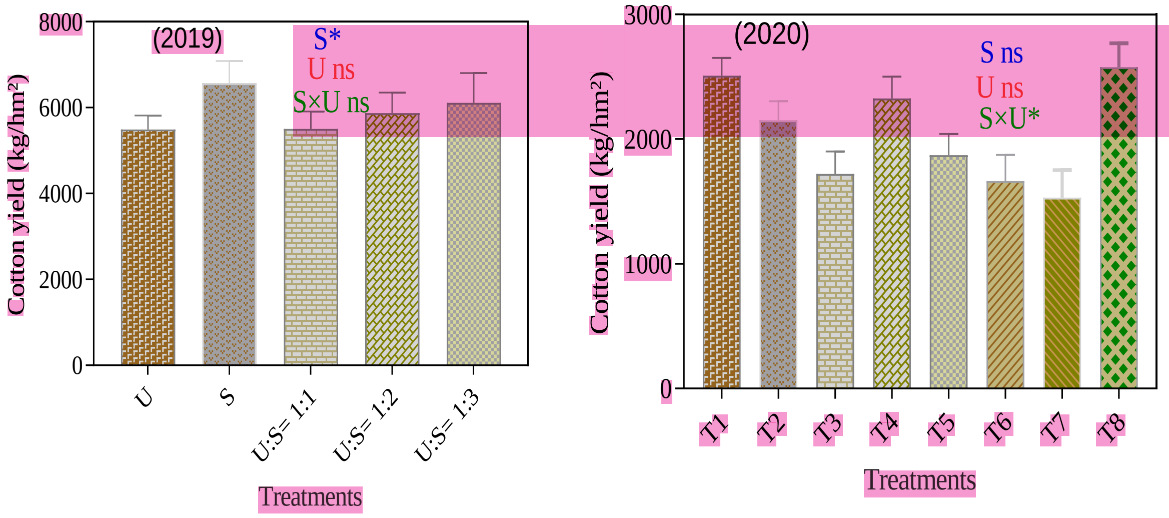

Figure 6. Effects of different irrigation methods on cotton yield: figure (2019) represents Experiment I mixing ratio irrigation method; figure (2020) represents Experiment II Round irrigation method. Data were presented as the mean ± standard deviation (SD) of three replicates at a significance level of $p < 0.05$ (based on two-way ANOVA).

Supplementry figure

Click here to access/download
Supporting Information
Supplementry fig.docx

Type of contribution: Research Article

Number of text pages: 1718

Number of tables: 8

Number of figures: 6

Title:

Effect of surface water and underground water drip irrigation on cotton growth and yield under two different irrigation schemes

Nihal Niaz[1] and Cheng Tang[1]*

[1]College of Agronomy, Shihezi University, Shihezi City, 832003, Xinjiang Province, China.

***Correspondence:** tangcheng1983@163.com; Tel.: +86 15352608029

Both authors contributed equally to this work

ABSTRACT: Two field experiments were conducted to investigate the effects of surface water and underground water drip irrigation on cotton yield, dry matter accumulation, and nutrients uptake under different irrigation regimes.,. The first experiment comprised of five ratios of underground water to surface water including; 1:0 (U), 0:1 (S), 1:1 (T_3), 1:24 (T_4) and 1:3 (T_5). While, the second experiment included comprised of eight treatments including: (i) 1:3 {U:S (T_1)}, (ii) 2:2 {U:S (T_2)}, (iii) 3:1 {U:S (T_3)}, (iv) 1:3 {S:U (T_4)}, (v) 2:2 {S:U (T_5)}, (vi) 3:1 {S:U (T_6)}, (vii) 4:0 {U:S (T_7)} and (viii) 0:4 {U:S (T_8)}. T1 {(1:3) underground water : surface

Formatted: Subscript

Formatted: Subscript

Formatted: Subscript

Formatted: Subscript

Formatted: Subscript

Formatted: Subscript

Formatted: Subscript

Formatted: Subscript

~~water}; (ii) T2 {U:S (2:2)}; (iii) T3 {U:S (3:1)}; (iv) T4 {S:U (3:1)}; (v) T5 {S:U (2:2)}; (vi) T6 {S:U (1:3)}; (vii) T7 {U:S (4:0)}; and (viii) T8 {U:S (0:4)}.~~ The average concentration of leaves dry matter after 8th irrigation significantly increased by 131.2% (S), 34.4% (U: S= 1:1), 59.3% (U: S= 1:2), and 93.7% (U: S= 1:3), respectively, compared with U treatment. Likewise, the stem dry matter increased from 48.5 g (U), to 122.2 g (S) and 101.6 g (U:S= 1:3). Furthermore, the results of two-way ANOVAs revealed a significant main and interactive effect on dry matter accumulation of cotton plants~~at various growth stages~~ treated with surface water at growth, boll and boll opening stages ~~in round irrigation scheme~~ (*p* < 0.05). For instance, the highest leaves dry matter in round irrigation at various growth stages were recorded in T8 treatment. Compared with T7 treatment an average increase rate of 50.6 % (growth), 100.9 % (boll) and 93.3 % (boll opening), in stem dry matter were recorded in T8 treatment. The soil available N at 0-20 cm after 8th irrigation recorded an average increase rate of 40.1%, 6.6%, 13.5%, and 29.5%, respectively, while at 20-40cm an average increase rate of 37.4 %, 7.1%, 20.0%, and 21.9% were noted (*p* < 0.05). Moreover, the concentration of N in round irrigation at 0–20 cm at different growth stages were 83.3 mg/kg~~±2.8~~ (growth stage), 79.01 mg/kg~~±1.84~~ (boll stage), 96.16 mg/kg~~±3.83~~ (boll opening stage) in T8, while in T7 the concentration of N was 36.1 mg/kg~~±5.9~~ (growth), 54.51 mg/kg~~±2.81~~ (boll), and 53.9 mg/kg~~±3.83~~ (boll opening) (*p* < 0.05). The highest cotton yield of 6571 kg/hm^2 was observed in S treatment compared with the U treatment (5492 kg/hm^2, U: S= 1:1 (5502 kg/hm^2), U: S= 1:2 (5873 kg/hm^2) and U: S= 1:3 (6111 kg/hm^2). Similarly, in round irrigation the increasing trend follow the order of T8 > T1 > T4 > T2 > T5 > T3 > T6 > T7. Overall, our findings provide useful information to current irrigation practices in water scarce regions. Alternatively, improving water use efficiency is a viable solution to the water scarcity. Therefore, surface water irrigation is recommended as an effective irrigation strategies to

Formatted: Font: Italic

Formatted: Font: Italic

Formatted: Font: Italic

improve cotton yield and growth.

~~Therefore, it is suggested that surface water irrigation should be applied to maximize the cotton yield, dry matter accumulation, nutritional status and overall growth of cotton crop.~~

Keywords:~~;;;~~ Irrigation strategies; Gossypium hirsutum L; dry matter accumulation; N~~,P,K~~, P, K uptake;~~;~~ underground water.

1. Introduction

Water for ~~I~~irrigation ~~water~~ is one of the most limiting factors for future global agricultural development [1, 2]. Climate change, and over-exploitation of water resources in ~~A~~arid and semi-arid regions of the world are being subjected to severe water shortages ~~due to climate change and over-exploitation of water resources~~ [3]. For instance, ~~Taken China is an example~~, in China the arid and semi-arid area occupies 52.5% of China's land area which is mainly distributed in Northwest, Northeast, and North China [4]. To achieve high grain yields ~~Agriculture sectors~~ in these regions ~~are totally dependent on~~ sufficient irrigation is required, as there are no reliable surface water sources for irrigation, groundwater is the main source of water for irrigation. Moreover, ~~T~~to fulfill the inherent irrigation water requirements, about 60% of the water resources including both surface and underground water is used for agriculture purposes. On the other hand ~~contrast~~, due to significant increase in population, there is competition for fresh water among municipal, industrial and agricultural sectors in several countries in the world especially in China [5]. Consequently, the allocation of fresh water to agriculture sector in these regions substantially decreased [6]. Therefore, improving irrigation efficienc~~y~~ies by introducing new irrigation strategies is the key and effective way to solve the problem of water shortage, ecological environment deterioration, and to develop agricultural production system in arid and

semi-arid regions.

Cotton (*Gossypium hirsutum* L.), an important source of natural fibers for to textile industries that serve the humanity from at least more than four to seven thousand years ago [7]. The current global cotton fiber production is estimated to be 24.65 million tons, with 6.71 million tons produced in America, 0.38 million tons produced in Europe, and 15.06 million tons produced in Asia [8]. Following this, only China accounts for one-quarter of the world's cotton output and one-third of the world's cotton consumption [9]. On the flip sideHowever, cotton production is completely dependent on irrigation i.e., the shortage of irrigation water resources restricts the comprehensive improvement of cotton productivity [10, 11]. On average, Wwater requirements for cotton growth vary from 700 to 1200 mm during the growing season, depending on irrigation method, and production goals [12]. Numerous studies have been conducted to examine water-use efficiency of cotton in recent years. For instance, a study conducted by Grismer [13] found noted that, in Arizona counties, for upland cotton actual evapotranspiration (ETc) water-use efficiency varied from 1.27 to 1.38 kg/ha-mm while, for pima cotton, its varied from 0.9 to 1.09 kg/ha-mm. In California counties, ETc water-use efficiency varied from 1.34 to 2.10 kg/ha-mm and 1.51–1.77 kg/ha-mm for upland and pima varieties, respectively. Furthermore, due to unevenly distribution of annual precipitation across the season, Bboth surface water resources (water from rivers and reservoirs) and groundwater resources (water stored in aquifers) were used for agriculture purposes in arid and semi-arid regions. However, irrigation with underground water has a negative impact on cotton productivity, plant nutritional condition, and dry matter accumulation. For instance, excessive underground water irrigation exacerbates the soil salinization problems, and reduce crop yield [11, 14]. Likewise, well water irrigation with low temperature potentially inhibits the growth and

development of cotton plant [15]. Moreover, low soil temperatures may slow down the uptake rate of nutrients such as N so much that they turn out to limit vegetative growth rates [16]. Following this Aat 20°C-RZT, nutrient concentrations have been significantly affected plant growth indexes, indicating that low root temperature inhibited high nutrient effects on plant growth [16, 17]. While on the other hand, surface water irrigation shows promising effect on increasing crops yield [18, 19]. For example, irrigating seedlings with warm water (surface water) can increase the stem thickness, leaf area, root coefficient, photosynthetic rate, dry matter per unit fresh weight, root-shoot ratio, and strong seedling index of seedlings [16]. Therefore, clarifying the effect of surface water and underground water on cotton growth and yield by applying different irrigation regime is of paramount importance for improving crop productivity by using less waterunder limited water supply.

Xinjiang Uygur Autonomous Region is one of the most water-scarce states in China. According to the Statistics Bureau of Xinjiang Uygur Autonomous Region, the water production per unit area of Xinjiang Uygur Autonomous Region is 51 mm, which is the second highest rank in the country [19]. The large number of glaciers in Xinjiang Uygur Autonomous Region and the small unit area are important issues of water resources in Xinjiang Uygur Autonomous Region. Snowmelt water from glaciers accounts for more than 25% of total surface water [20]. However, according to China's statistics, Xinjiang's total cotton output in 2017 was 4.082 million tons, accounting for 74.4% of the country's total production [20]. While on the other side, agriculture in Xinjiang is totally dependent on irrigation, according to 2016 Xinjiang Water Resource Bulletin, agriculture consumes 94.3% of the available total water resources.

In the present-day context, lot of emphasis is being given on improvements in irrigation practices to increase crop production and to sustain the productivity levels. In this study, two

field experiments were carried out in calcareous soils. We hypothesize that surface water drip irrigation outcompetes underground water irrigation in increasing cotton yield, dry matter accumulation and NPK uptake. Therefore, the objectives of our study were to (i) compare the effects of different irrigation schemes on cotton growth and yield. (ii) Clarify the response of cotton growth to surface and underground water addition, (iii) finally put forward an appropriate irrigation strategy to increase cotton yield in calcareous soil.

2. Materials and Methods

Soil properties	0-20cm

Formatted Table

2.1 Experimental site

The study area is located in the Mosuowan reclamation region (44° 03′ N, 86° 05′ E), which is located on the northern slope of Tianshan Mountain in Xinjiang and is surrounded by the Gurbantunggut Desert. According to WRB soil taxonomy, the tested soil is classified as Calcisol Fluvisols. The study area has a typical continental climate with a mean annual precipitation of 115 mm, and rainfall largely occurs from April to July. The mean annual potential evapotranspiration is approximately 2,000 mm. The physicochemical characteristics of given soil is listed in (Table 1).

Table 1. Selected physical and chemical properties of the tested soils.~~**Insert "(Table 1)"**~~

Data were presented as the mean ± standard error (SE), n=3 at a significance level of $p < 0.05$.
a. pH was determined at soil to milli-Q water ratio of 1:5 w/v using pH meter.
b. Organic matter was measured by potassium dichromate volumetric method (Shaw, 1959) [37].
c. Total N was measured by the semimicro-Kjeldahl method (Bao 2000).
d. Total P was measured by the perchloric acid digestion method (Bao 2000).
e. Total and available K was measured by the flame photometry method (Bao 2000).

Formatted

pH	8.83±0.03
OM (g kg^{-1})	13.7±0.23
Total-N (g kg^{-1})	1.20±0.05
Available-P (mg kg^{-1})	17.5±3.52
Total-P (g kg^{-1})	1.08±0.12
Total-K (mg kg^{-1})	21.0±2.07
Available-K (mg kg^{-1})	241.4±0.00

Formatted: Justified

2.2 Experimental Design

The first experiment (different mixing ratio) was a randomized complete block design (RCBD) field experiment conducted on April 12, 2019 with five treatments including U (underground water); S (surface water); U:S= 1:1; U:S= 1:2; U:S= 1:3, respectively. Each treatment was replicated three times while the area of each block was 55.2 m^2. For the mixing ratio experiment we dig several whole and cover it with plastic, after that we supply surface and underground water into the whole with a constant water ratio by using water ratio measurement instrument. After supplying water we mix the nutrient with the water and supply it to cotton field through drip irrigation. The schematic of our experimental design are presented in supplementary figure 1. The supply of water and nutrients for each irrigation has listed in (Table 2).

Insert ~~" Supplementary~~"Supplementary figure 1 and (Table 2)"

Table 2. The amount of nutrients (fertilizer) and water during the period of cotton irrigation (mixing ratio irrigation 2019).

**Irrigation time**	**Irrigation water (m³.hm⁻²)**	**Nitrogen (Kg.hm⁻²)**	**Phosphorus (Kg.hm⁻²)**	**Potassium (Kg.hm⁻²)**
12.6	450	30	15	15
20.6	450	75	75	75
30.6	375	105	105	105
10.7	375	105	105	105
20.7	300	75	150	150
31.7	300	30	180	180
10.8	225	75	75	75
24.8	225	45	45	45

Formatted

The second experiment (round experiment) was a field randomized complete block design (RCBD), comprised of ~~having~~ eight treatments ~~consisted of~~including, 1:3 (T_1), 2:2 (T_2), 3:1 (T_3), 1:3 {S:U (T_4)}, 2:2 {S:U (T_5)}, 3:1 {S:U (T_6)}, 4:0 (T_7) and 0:4 (T_8). ~~U:S=1:3, U:S =2:2, (iii) T3 {U:S (3:1)}; (iv) T4 {S:U (3:1)}; (v) T5 {S:U (2:2)}; (vi) T6 {S:U (1:3)}; (vii) T7 {U:S (4:0)}; and (viii) T8 {U:S (0:4)}.~~ The treatment ratios represents the supply of surface and underground water in different stages of cotton growth i.e., seedling stage, growth stage, boll stage and boll opening stage. In round water irrigation we supply the constant ration of both surface water and underground water directly to the cotton field at various growth stages without mixing it. For maintaining the required ratios we first supply the specific ratio of surface water and then we supply the specific ratio of underground water by using a water ratio measurement meter. Further details about irrigation is given in (Table 3). Nutrients (fertilizer) and water were supplied through drip irrigation. The supply of water and nutrients for each irrigation has listed in (Table 4).

Insert "(Table 3 and 4)"

Table 3. Supply of surface and underground water at different growth stage of cotton (round irrigation 2020)

**Treatments**	**Seedling stage**	**Growth stage**	**Boll stage**	**Boll opening stage**
T1 U:S (1:3)	[1]U	S	S	S
T2 U:S (2:2)	U	U	S	S
T3 U:S (3:1)	U	U	U	S
T4 S:U (3:1)	[2]S	S	S	U
T5 S:U (2:2)	S	S	U	U
T6 S:U (1:3)	S	U	U	U
T7 U:S (4:0)	U	U	U	U
T8 U:S (0:4)	S	S	S	S

[1] U represents the supply of underground water.
[2] S represents the supply of surface water.

Formatted: Justified

Formatted: Justified

Table 4. The supply of water and nutrients (fertilizer) for each irrigation (round irrigation 2020).

Irrigation order	Water ($m^3/667m^2$)	Nitrogen ($Kg/667m^2$)	Phosphorus ($Kg/667m^2$)	Po[illegible] ($Kg/667m^2$)
1	30	2	1	1
2	30	5	5	5
3	25	7	7	7
4	25	7	7	7
5	20	5	10	10
6	20	2	12	12
7	15	5	-	-
8	15	3	-	-

2.2 Soil and Plants sampling

Soil samples were collected at depths of 0-20 cm and 20-40 cm from each block after 3-5 days of irrigation. Samples were air dried, sieved through 1mm and 0.15 mm for nutrients determination. The pH was determined at soil to milli-Q water ratio of 1:5 w/v using pH meter. The organic matter was determined by potassium dichromate volumetric method. Nitrogen was determined by semimicro Kjeldahl method [37]. Phosphorus was measured by the perchloric acid digestion method [38]. Potassium was determined by flame photometry [37]. Soil available Nitrogen, soil available phosphorus, soil available potassium and soil organic matter were determined in soil samples after growth stage, boll stage and boll opening stage.

Plant samples were randomly collected from each block at interval of 3-5 days after each irrigation. Plant samples were divided into the following parts (leaves, stems, roots and fruits), washed with tap water and then dried in oven at 105˚C for 30 minutes and then at 75˚C for 3 days. The plant samples were then weighed with balance and the dry matter data were calculated after growth stage, boll stage and boll opening stage, following the method of [39] (Figure 1).

Insert "(Figure 1)"

2.3 Experimental Design

The first experiment was a randomized complete block design field experiment done on April 12, 2019 with five treatments including U (underground water); S (surface water); U:S= 1:1; U:S= 1:2; U:S= 1:3, respectively. Each treatment was replicated three times while the area of each block was 55.2 m^2. The supply of water and nutrients for each irrigation has listed in (Table 2).

Insert "(Table 2)"

The second experiment was a field randomized complete block design (RCBD) having eight treatments consisted of U:S=1:3, U:S =2:2, (iii) T3 {U:S (3:1)}; (iv) T4 {S:U (3:1)}; (v) T5 {S:U (2:2)}; (vi) T6 {S:U (1:3)}; (vii) T7 {U:S (4:0)}; and (viii) T8 {U:S (0:4)}. The treatment ratios represents the supply of surface and underground water in different stages of cotton growth i.e., seedling stage, growth stage, boll stage and boll opening stage. Further details about irrigation is given in (Table 3). Nutrients (fertilizer) and water were supplied through drip irrigation. The supply of water and nutrients for each irrigation has listed in (Table 4).

Insert "(Table 3 and 4)"

2.3 Soil and Plants sampling

Soil samples were collected at depths of 0-20 cm and 20-40 cm from each block after 3-5 days of irrigation. Samples were air dried, sieved through 1mm and 0.15 mm for nutrients determination. The pH was determined at soil to milli-Q water ratio of 1:5 w/v using pH meter. The organic matter was determined by potassium dichromate volumetric method. Nitrogen was determined by semi micro-Kjeldahl method [37]. Phosphorus was measured by the per chloric acid digestion method [38]. Potassium was determined by flame photometry [37]. Soil available Nitrogen, soil available phosphorus, soil available potassium and soil organic matter were

determined in soil samples after growth stage, boll stage and boll opening stage.

Plant samples were randomly collected from each block at interval of 3-5 days after each irrigation. Plant samples were divided into the following parts (leaves, stems, roots and fruits), washed with tap water and then dried in oven at 105˚C for 30 minutes and then at 75˚C for 3 days. The plant samples were then weighed with balance and the dry matter data were calculated after growth stage, boll stage and boll opening stage, following the method of [39] (Figure 1).

Insert "(Figure 1)"

2.4 Statistical analysis

Data were analyzed using the SPSS 25.5 statistical program (SPSS Inc., Chicago, IL, USA) with two-way ANOVA at a significance level of $p < 0.05$. A Duncan multiple range test was carried out to test the significant differences between different treatments. GraphPad Prism 12.0 software (GraphPad Software, Inc., San Diego, CA, USA) was used for data processing and images making. All results in figures and tables were presented as mean ± standard deviation (SD) of three replicates, and a significance level of P p <0.05 was used for all analysis.

Formatted: Font: Italic

Formatted: Font: Italic

3. Results

3.1 Effects of different mixing ratio irrigation method on the dry matter accumulation

The average dry matter accumulation in the different parts of the cotton plant fluctuated greatly with different irrigation treatments and increased sharply after each irrigation time (Figure. 2). For instance, the significant maximum dry weights (DW) were recorded in S treatment followed by the U: S= 1:3 treatment. However, the lowest dry matter accumulation was recorded in the U treatment. The concentration of leaves dry matter after 8th irrigation increased from 32.8 ± 0.15 g (U), to 74.1 ± 0.27g (S), 43.5 ± 0.18g (U:S= 1:1), 51.1 ± 0.21g

(U:S= 1:2) and 62.9 ± 0.37g (U:S= 1:3), with an average increase rate of 131.2%, 34.4%, 59.3%, and 93.7%, respectively ($p < 0.05$, Figure 2a). Compared with underground water the tremendous average increase rate of 131.2% and 93.7% were noted in surface water and different mixing ratios. Similarly, stem dry matter of five applied treatments as affected by eight irrigation regimes are presented in Figure 2b. The concentration of stem dry matter after 8th irrigation increased from 48.5 g (U), to 122.2 g (S) and 101.6 g (U:S= 1:3), with an average increase rate of 151.5 % and 109.5%, (Figure 2b). Moreover, compared with underground water irrigation treatment (24.6 g), a significant highest root dry matter was recorded with surface water treatment (51.74 g) and U: S= 1:3 treatment (46.96 g) ($p < 0.05$, Figure 2c). Similarly, compared with surface water, underground water significantly suppressed roots dry matter accumulation and follows the sequence of S> U: S= 1:3> U: S= 1:2> U: S= 1:1>U. The highest fruits dry matter accumulation was recorded in the treatment of surface water (83.43 ± 0.34 g) compared with all other treatments ($p < 0.05$, Figure 2d).

Insert "(Figure 2)"

Formatted: Font: Italic

Formatted: Font: Italic

Formatted: Font: Italic

3.2 Effects of round irrigation method on dry matter accumulation

Round irrigation method also significantly affected dry matter accumulation in the different parts of the plant at various stages of growth ($p < 0.05$, Figure. 3). The biomass of yield-related organs showed a trend of gradual increase with the progression of the growth period, and the most intense change was at the beginning of the boll development stage. Maximum dry weights (DW) were achieved at boll opening stage and minimum at growth stage. The DW of leaves, stem, roots and fruits continued to increase initially and then declined and stayed stable comparatively because of leaf senescence and termination of reproductive development at final

Formatted: Font: Italic

stages (Figure 3a-d). The highest leaves dry matter of 14.4g (growth stage), 88.8g (boll stage) and 100.7g (boll opening stage) were observed in T8 treatment compared with the lowest, 8.9g (growth stage), 42.9g (boll stage) and 78.7g (boll opening stage) of T7 treatment. Results of Two-way ANOVAs reveals a significant main and interactive effect on various stage of leaves dry matter treated with surface water ($p < 0.05$, Figure. 3a). Compared with T7 treatment an average increase rate of 50.6 %, 100.9 % and 93.3 %, at growth, boll and boll opening stage respectively in stem dry matter were noted in T8 treatment. Likewise, a significant main and interactive effect were observed at various stages of stem dry matter ($p < 0.05$, Figure. 3b). Also, it was evident from the results that the highest root dry matter recorded in growth, boll and boll opening stage were 6.46g, 38.68g and 51.63g with T8 treatment, compared with the lowest values of 4.60g, 12.24g and 23.57g recorded in T7. A significant main and interactive effect were observed at boll and boll opening stages of roots dry matter ($p < 0.05$, Figure. 3c). Fruits dry matter were produced in boll and boll opening stages and was substantially higher in T8 treatment compared with other applied treatments throughout the whole experiment. A significant main interaction effect on boll and boll opening stage while a significant interactive effect on boll stage were observed for fruits dry matter ($p < 0.05$, Figure. 3d). Our result showed that the surface water irrigation along with mixing ratio shows promising effect on average dry matter accumulation in cotton plant.

Formatted: Font: Italic

Formatted: Font: Italic

Formatted: Font: Italic

Formatted: Font: Italic

Insert "(Figure 3)"

3.3 Effects of different mixing ratio and round irrigation on soil available nutrients

Soil available nitrogen at a depth of 0–20 and 20–40 cm fluctuated greatly during the whole cotton growth period, and increased sharply after each irrigation time (Figure. 4a, b). The

concentration of N at 0–20 cm after 8th irrigation increased from 72.3 ± 0.15 mg/kg (U), to 101.3 ± 0.27 mg/kg (S), 77.1 ± 0.18 mg/kg (U:S=1:1), 82.1 ± 0.21 mg/kg (U:S=1:2) and 99.3 ± 0.37 mg/kg (U:S=1:3), with an average increase rate of 40.1%, 6.6%, 13.5%, and 29.5%, respectively ($p < 0.05$, Figure 4a). Compared with U treatment an average increase in N concentration at 20–40 cm after 8th irrigation in S treatment were 37.4 %, 7.1%, 20.0%, and 21.9% ($p < 0.05$, Figure 4b). Trends of soil available nitrogen follows the order of S> U:S=1:3> U:S=1:2> U:S=1:1>U. Moreover, the accumulation of P in different soil depths 0–20 and 20–40 cm was presented in (Figure. 4c, d). The concentration of P at 0–20 cm after 8th irrigation increased by 76.9%, and 33.8% %, respectively in S and U:S=1:3 treatments compared with U treatment ($p < 0.05$, Figure 4c). Whilst at 20-40 cm, the amount of P in surface water application significantly increased from 12.6 ± 0.15 mg/kg (U), to 28.4 ± 0.27 mg/kg (S), 13.4 ± 0.18 mg/kg (U:S=1:1), 13.3 ± 0.21 mg/kg (U:S=1:2) and 22.6 ± 0.37 mg/kg U:S=1:3 ($p < 0.05$, Figure 4d). The status of available potassium in soil at 0–20 and 20–40 cm remains parallel throughout the experiment (Figure 5a, b). Compared with other treatments the surface water treatment shows maximum concentration of potash, however the difference between different applied treatments were negligible. Soil organic matter was significantly affected by different irrigation treatments (Fig. 5c, d). However, at the start of the experiment the S treatment followed by U: S=1:3 treatment shows potential promising effect at both 0–20 and 20–40 cm, while the difference between other treatments were negligible.

Formatted: Font: Italic

Formatted: Font: Italic

Formatted: Font: Italic

Formatted: Font: Italic

Furthermore, the concentration of soil available nutrients at a depth of 0-20cm and 20-40 cm in round irrigation method significantly affected by different applied treatments. The concentration of N recorded at 0–20 cm at different growth stages were 83.3±2.8 (growth stage), 79.01±1.84 (boll stage), 96.16±3.83 (boll opening stage) in T8, while in T7 the concentration of

N was 36.1±5.9 (growth), 54.51±2.81 (boll), and 53.9±3.83 (boll opening) ($p < 0.05$, Table 5). The soil available P content significantly increased in the initial and final stages with T8 treatment throughout the experiment ($p < 0.05$, Table 6). While, the status of available potassium in soil remains parallel throughout the experiment, the difference between different applied treatments were negligible ($p < 0.05$, Table 7). However, the maximum concentration of potash was determined in T8 treatment. Soil organic matter was significantly affected by different irrigation treatments ($p < 0.05$, Table 8). However, at the start of the experiment the surface water irrigation treatment shows promising effect at both 0–20 and 20–40 cm, while the difference between other treatments were negligible.

Formatted: Font: Italic

Formatted: Font: Italic

Formatted: Font: Italic

Formatted: Font: Italic

Insert "(Figure 4 and Figure 5)"

Insert "(Table 5, Table 6, Table 7 and Table 8)"

Table 5. Effect round irrigation method on soil available nitrogen (mg/kg). Data were presented as the mean ± standard deviation (SD) of three replicates at a significance level of p < 0.05 (based on ANOVA).

Table 6. Effect round irrigation method on soil available phosphorus (mg/kg). Data were presented as the mean ± standard deviation (SD) of three replicates at a significance level of p < 0.05 (based on ANOVA).

Soil depth	Treatments	Growth stage	Boll stage	Boll opening stage
0-20 cm	1	77.8±3.8a	77.18±1.84a	78.4±3.83b
	2	59.4±4.6b	75.34±1.84a	75.34±4.86b
	3	37.4±3.8d	67.38±2.81b	60.03±3.83cd
	4	44.7±3.8c	74.11±4.62a	63.09±4.62c
	5	39.8±2.8d	67.38±3.83b	61.25±2.81cd
	6	37.1±2.3d	67.38±1.06b	57.58±3.83cd
	7	36.1±5.9d	54.51±2.81c	53.9±3.83d
	8	83.3±2.8a	79.01±1.84a	96.16±3.83a
20-40 cm	1	55.7±3.8a	50.84±2.81a	52.37±1.3ab
	2	45.9±1.8b	34.91±1.84b	53.9±2.81a
	3	28.2±1.1c	27.56±4.86bc	44.1±1.84abc
	4	29.7±2.3c	33.69±3.83b	47.78±4.86ab
	5	28.2±2.8c	29.4±5.51bc	47.78±1.84ab
	6	22.4±8cd	24.5±2.81c	41.65±3.83bc
	7	17.2±3.8d	23.89±4.86c	33.69±7.65c
	8	60.6±4.9a	52.68±4.62a	47.16±12.23ab

Soil depth	Treatments	Growth stage	Boll stage	Boll opening stage
0-20 cm	1	30.89±0.27ab	30.23±0.45b	28.36±0.22b
	2	29.98±0.58ab	28.33±0.44c	28.01±0.29b
	3	22.56±1.68c	23.14±0.25f	20.13±0.21e
	4	25.72±6.69bc	27.52±0.21d	23.75±0.12c
	5	22.91±4.52c	25.3±0.49e	22.36±0.93d
	6	20.51±4.61c	19.47±0.55g	19.18±0.3f
	7	20.23±4.06c	19.11±0.37g	18.18±0.19g
	8	34.44±1.67a	31.18±0.2a	31.52±0.37a
20-40 cm	1	19.33±0.69a	18.64±0.07ab	16.69±0.36b
	2	17.84±5.36a	18.03±1.65ab	14.95±0.3c
	3	12.13±0.62bc	17.04±0.3bc	13.44±0.29d
	4	16.79±0.63ab	17.93±0.3ab	14.52±0.28c
	5	17.88±2.49a	17.18±1.95bc	14.47±0.53c
	6	11.48±2.32c	15.93±0.63c	11.12±0.42e
	7	9.72±4.77c	12.66±0.29d	11.17±0.62e
	8	19.69±0.81a	19.11±0.39a	18.15±0.22a

Formatted: Justified

Table 7. Effect round irrigation method on soil available potassium (mg/kg). Data were presented as the mean ± standard deviation (SD) of three replicates at a significance level of $p < 0.05$ (based on ANOVA).

Soil depth	Treatments	Growth stage	Boll stage	Boll opening stage
0-20 cm	1	471.42±2.4b	481.32±2.51a	451.62±3.07b
	2	457.99±2.58c	473.16±5.52b	433.71±2.05c
	3	418.3±3.07f	464.16±2.84c	418.35±3.34e
	4	442.24±2.83d	470.73±2.56b	429.47±1.43c
	5	431.85±1.84e	469.95±1.25b	423.96±4d
	6	400.64±2.07g	462±2.17c	408.89±3.04f
	7	368.54±5.57h	429.11±2.35d	373.68±1.54g
	8	496.89±2.7a	483.81±2.73a	473.05±3.19a
20-40 cm	1	401.43±2.58b	376.18±8.94a	406.25±1.89b
	2	368.45±1.46c	367.14±0.1b	386.44±3.22c
	3	346.49±2.47f	355.5±4.22c	361.91±3.15f
	4	362.91±2.31d	365.74±1.57b	377.18±2.32d
	5	355.72±3.57e	360.36±3.52bc	370.62±3.39e
	6	333.71±2.68g	332.15±4.61d	346.2±2.94g
	7	322.19±3.16h	324.94±3.11d	324.11±1.36h
	8	434.53±3.14a	383.16±3.84a	428.69±2.04a

Formatted: Justified

Table 8. Effect round irrigation method on soil organic matter (g/kg). Data were presented as the mean ± standard deviation (SD) of three replicates at a significance level of $p < 0.05$ (based on

ANOVA).

Formatted: Justified

Soil depth	Treatments	Growth stage	Boll stage	Boll opening stage
0-20 cm	1	13.3371±0.33569b	12.4866±0.68639ab	13.1195±0.11468a
	2	13.0145±0.58669bc	11.0622±0.35745abc	12.0848±0.09785b
	3	11.7308±0.36465ef	10.5849±0.31272c	11.2333±0.95552bc
	4	12.5637±0.32678cd	10.9999±0.64795abc	11.5946±0.31635bc
	5	12.2023±0.4165de	10.7173±0.41908bc	11.3057±0.30968bc
	6	11.4874±0.43846ef	10.252±0.71663c	11.1814±0.83826bc
	7	11.3291±0.37229f	10.2181±0.24148c	10.8893±0.12844c
	8	14.4407±0.2702a	12.6024±2.42886a	13.427±0.36673a
20-40 cm	1	10.6436±0.28389a	9.868±0.28809a	10.6518±0.2795a
	2	10.4489±0.13549ab	9.687±0.22901a	10.4804±0.32128a
	3	9.9319±0.71469ab	9.3676±0.16334a	9.7692±0.25222b
	4	10.1805±0.09637ab	9.5965±0.53775a	9.9897±0.22095b
	5	10.1261±0.41629ab	9.4632±0.52158a	9.9327±0.27535b
	6	9.458±0.22105bc	9.1858±0.38905a	9.6588±0.11612b
	7	8.6652±0.77305c	9.1339±0.53729a	9.6424±0.21155b
	8	10.7595±0.99849a	9.911±0.37617a	10.7889±0.2811a

3.4 Effects of different irrigation methods on cotton yield

The effect of different mixing ratio irrigation on cotton yield is presented in Figure 6a. Cotton yield responded significantly to different water irrigation modes. For instance, the maximum cotton yield 6571 kg/hm^2 was observed in (S) treatment compared with the treatment (U) (5492 kg/hm^2), U: S= 1:1 treatment (5502 kg/hm^2), U: S= 1:2 treatment (5873 kg/hm^2) and U: S= 1:3 treatment (6111 kg/hm^2). Likewise, round irrigation potentially effect cotton yield under different applied treatments (Figure 6b). For example, the increasing trend follow the order of T8 > T1 > T4 > T2 > T5 > T3 > T6> T7, which shows that surface water irrigation can effectively guarantee cotton production. The highest cotton yield was observed in T8 treatment in which surface water was supplied through all stages of cotton growth, followed by treatment T1 and T4 (Figure 6b). However, the lowest yield was recorded in treatment T7 in which under-

ground water was supplied.

Insert "(Figure 6)"

4. Discussion

Water for irrigation is a major limitation to agricultural production in the Xinjiang region. The Xinjiang region in Northwest China is one of the most important cotton producers with the . The cotton plantation area ofin Xinjiang is about 1.8 × 106 ha, accounting for 54% of China's total cotton planting area [20]. Approximately, aboutIt produced 451 × 104 tons of cotton were produced (in 2014, accounting for 73% of China's total cotton production [18]. Meanwhile, surface water evaporation caused by high temperatures results in a severe water shortage in southern Xinjiang leading to soil salinization, a lowered survival rate for crops, and slow development of local agriculture [21]. Similarly, increased usage of underground water for irrigation exacerbates the soil salinization problems, which significantly reduce crop yield [10, 11]. In this study, the application of surface water in both mixing ratio and round irrigation methods, significantly promotes dry matter accumulation (Fig. 2-3), NPK uptake (Fig. 4,5 and Table 5-8) and cotton yield (Fig. 6), compared with all other applied treatments. Our obtained results are in line with the findings of previous published literature [34, 35, 36]. The superiority of surface water over underground water in calcareous soil may likely be due to the following reasons; In line with the previous findings, in these experiments surface water application in both round and mixing ratio irrigation methods, significantly promotes dry matter accumulation (Fig. 2-3), NPK uptake (Fig. 4,5 and Table 5-8) and cotton yield (Fig. 6), compared with all other applied treatments. The most likely reasons may be, in general surface water possess high temperature while the underground water temperature is quite low. Previous study has shown

that well water irrigation with low temperature potentially inhibits the growth and development of jujube [22]. Likewise, several published literatures have shown that underground water irrigation (low temperature water) affect the growth, yield, dry matter accumulation and active developmental stages of grains plants such as peanuts, cucumber, and tomato [23, 24, 25, 26]. For instance, a study conducted by Meng et al. (2016), noted that underground water irrigation significantly affects the growth and development of cotton plant. Similar results with the application of underground water irrigation is also obtained in this study. Furthermore, Deng et al., [27], also pointed out that underground water irrigation along with their low temperature properties significantly retarded the growth of vegetables and their photosynthetic developments. Consequently, the excessive usage of underground water irrigation results in the accumulation of toxic substances in soil which alternatively leads to reduction in plants and grains yields [28, 29]. The accumulation of salt can directly decrease soil nutrient efficiency by inhibiting microbial mineralization activity in soil [30]. Additionally, salinity can also indirectly affect soil nutrient cycling and efficiency by destroying soil physical structure [31, 32, 33]. On the flip side, ~~T~~the study of Z~~z~~hang et al., 2002 [34] showed that alternate irrigation with surface fresh water can reduce soil salt content and increase cotton production which are in line with our findings. In this study surface water irrigation along with ~~the~~ different mixing ratios irrigation also shows promising effects on cotton yield and dry matter accumulation when compared with underground water treatment alone, this is possibly due to when the underground water and surface water were mixed together, the temperature and salt content were changed. For example, a study conducted by Tao et al. 2014 [35], showed that mixed irrigation mode of brackish and fresh water with a salinity of 1.6g/L could achieve higher crop yield with better quality. Consistently, a study carried out by Wang et al. 2010 [36], showed that well and canal mixed irrigation could

keep the salt balance of root soil even in relatively dry years, while well irrigation alone results in salt accumulation in roots of winter wheat and decreased the yield up to 20% -30%. All these findings suggest that under-ground water irrigation possess negative effects on plant yield and growth whilst sur-face water irrigation and different mixing ratios irrigation –significantly promote cotton yield NPK, uptake and dry matter accumulation.

5. Conclusions

It can be concluded that the application of surface water along with their different mixing ratios irrigation outcompete underground water irrigation in both mixing ratio and round applied irrigations methods. A significant highest The dry mater accumulation, nutrients uptake and cotton yield at various stages i.e., growth stage, boll stage, and boll opening stage were always noted significantly higher in surface water applied treatments compared with underground water treatment. Overall, our findings provide meaningful information to current irrigation practice in increasing cotton growth and yield. Therefore taken into account the scarcity of water resource We suggest that surface water irrigation application is recommended as an effective irrigation strategy in Xinjiang calcareous soil for better cotton yield and nutrient uptakes.

Disclosure statement

The authors declare that they have no conflict of interest.

Funding

This work was jointly supported by National Key Research and Development Program of China (2017YFC0504302-02), and the High-level introduction of talent research start-up project in Shihezi University (RCZK2018C20).

This research was jointly supported by National Natural Science Foundation of China (41161047), and the Twelfth Five-Year National Technology Support Program (2012BAD42B02).

References

1. UN-Water. The United Nations World Water Development Report 4: Managing Water under Uncertainty and Risk; United Nations World Water Assessment Programme, UNESCO: Paris, France, 2012.
2. Gu Z, Qi Z, Ma L, Gui D, Xu J, Fang Q, Yuan S, Feng G. Development of an irrigation scheduling software based on model predicted crop water stress. Comput. Electron. Agric. 2017; 143, 208–221.
3. Feike T, Ling YK, Mamitimin Y, Nan H, Lin L, Abdusalih N, Xiao H, Doloschutz R. Determinants of cotton farmers' irrigation water management in arid northwestern china. Agric. Water Manag. 2017; 187, 1–10.
4. Wei Qu, Yanmei Tan, Zhentao Li, Eefje Aarnoudse, Qin Tu. Agricultural Water Use Efficiency—A Case Study of Inland-River Basins in Northwest China. Sustainability. 2020; 12, 10192.
5. Meng B, Liu JL, Bao K, Sun B.Water fluxes of Nenjiang river basin with ecological network analysis: Conflict and coor-dination between agricultural development and wetland restoration. J. Clean. Prod. 2019; 213, 933–943.
6. Liu Y, Jiang H, Li C, Huang H, Pan Z, Chai C. Analysis of irrigation water requirement and irrigation requirement index for cotton of Hebei province. Nongye Gongcheng Xuebao/Trans. Chin. Soc. Agric. Eng. 2013; 29, 98–104.
7. Li Y, Gao X, Tenuta M, Gui D, Li X, Xue W, Zeng F. Enhanced efficiency nitrogen fertilizers were not effective in reducing N2O emissions from a drip-irrigated cotton field in arid region of Northwestern China. Sci. Total Environ. 2020; 748, 141543.
8. FAOSTAT. 2021. Available online: http://www.fao.org/faostat/en/#data/QCL. (accessed on 25 July 2021).
9. Ma C, Mamat S, Yao J, Isak G. Spatio-temporal changes of cotton production in China from 1950 to 2015. Acta Geogr. Sin. 2020; 75, 1699–1710.
10. Sorensen RB, Lamb MC, Butts CL. Crop rotation, irrigation system, and irrigation rate on cotton yield in southwestern Georgia. Crop. Forage Turfgrass Mgmt. 2020; 6, e20053.
11. Mahmoodi-Eshkaftaki M, Rafiee MR. Optimization of irrigation management: a multi-objective approach based on crop yield, growth, evapotranspiration, water use efficiency and soil salinity. Journal of Cleaner Production. 2020; 252, 119901.
12. Evett SR, Baumhardt RL, Howell TA, Ibragimov NM, Hunsaker DJ. Cotton. Crop Yield Response to Water; FAO ir-rigation and drainage paper. No. 66; FAO: Rome, Italy. 2012; pp. 152–161.
13. Grismer ME. Regional cotton lint yield, ETc, and water value in Arizona and California. Agric. Water Manag. 2002; 54, 227–242.

14. Sekhon KS, Kaur A, Thaman S, Sidhu A S, Garg N, Choudhary O P, Buttar G S, Chawla N. Irrigation water quality and mulching effects on tuber yield and soil properties in potato (*Solanum tuberosum* L.) under semi-arid conditions of Indian Punjab. Field Crops Research. 2020; 247, 107544.
15. Meng A. Effect of drip irrigation temperature on grey desert soil environment and cotton growth. Urumqi: Xinjiang Agricultural University. 2016; 39-40.
16. Kong Q. Analysis on the key factor of inhibiting cotton production development and discussion on its strategies. Xinjiang Agric. Sci. 2010; 47, 3–5 (in Chinese with English abstract).
17. Kang Yaohu, Wang Ruoshui, Wan Shuqin, Hu Wei, Jiang Shufang, Liu Shiping. Effects of different water levels on cotton growth and water use through drip irrigation in an arid region with saline ground water of Northwest China. Agric. Water Manag. 2013; 109, 117– 126.
18. Chen Z, Niua Y, Zhaoa R, Hana C, Hanb H, Luoa H. The combination of limited irrigation and high plant density optimizes canopy structure and improves the water use efficiency of cotton. Agric. Water Manag. 2019; 218, 139–148.
19. Wang J, Zhao S, Tan X, Liang Y, Gong Z, Ai X, Guo J, Maimaiti M, Li X, Zheng J. Production situation and cotton seed industry development report in Xinjiang cotton planting region in 2018. Cotton Sci. 2019; 41, 9–14.
20. Tian JS, Zhang XY, Yang YL, Yang CX, Xu SZ, Zuo WQ, Zhang WF, Dong HY, Jiu X L, Yu Y C, Zhao Z. How to reduce cotton fiber damage in the Xinjiang China. Industrial Crops and Products. 2017; 109, 803-811.
21. Fang S, Tu W, Mu L, Sun Z, Yang Y. Saline alkali water desalination project in southern Xinjiang of China: a review of desalination planning, desalination schemes and economic analysis. Renewable and Sustainable Energy Reviews. 2019; 113, 109268.
22. Zhang RW, Tian JC, Ma JM. The effect of different irrigation water temperatures on the growth and photosynthesis of leaf lettuce . China Rural Water and Hydropower. 2017; 4, 1 - 2.
23. Bai YY, Wang HD, Li M. et al. Effect on different irrigation water temperatures on tomato seedlings growth in greenhouse. Water Saving Irrigation. 2012; 11, 16 - 17.
24. Shi PX, Liu YR, Zhang XJ. et al. Effects of irrigation with low temperature water on soil enzyme activity and soil nutrient of peanut rhizosphere. Chinese Journal of Oil Crop Sciences. 2016; 38, 811 - 816.
25. Kuang W, Gao X, Gui D, Tenuta M, Flaten DN, Yin M, Zeng FJ. Effects of fertilizer and irrigation management on nitrous oxide emission from cotton fields in an extremely arid region of northwestern China. Field Crop Research. 2018; 229, 17-26.
26. Sun SJ, Cui SM, Song Y. et al. Effects of root zone temperature on growth and photosynthetic parameters of grafted cu-cumber. Agricultural Biotechnology. 2018; 7, 27 - 31.

27. Deng HL, Tian JC, Ouyang Z. Effect of Irrigation water temperature on soil temperature in vegetable root zone in greenhouse. Ningxia Engineering Technology. 2018; 17, 97 - 101.
28. Bumgaer NR, Scheerens JC, Mullen RW, et al. Root-zone temperature and nitrogen affect the yield and secondary me-tabolite concentration of fall and spring grown, high-density leaf lettuce. Journal of the Science of Food ＆ Agriculture. 2012; 92, 116 - 124.
29. Li LJ, Lu XC, Ma HY. et al. Comparative proteomic analysis reveals the roots response to low root-zone temperature in Malus baccata. Journal of Plant Research. 2018; 131, 865 - 878.
30. Rath KM, Rousk J. Salt effects on the soil microbial decomposer community and their role in organic carbon cycling: a review. Soil Biology Biochemistry. 2015; 81, 108-123.
31. Lakhdar A, Rabhi M, Ghnaya T, Montemurro F, Jedidi N, Abdelly C. Effectiveness of compost use in salt-affected soil. Journal of Hazardous Materials. 2009; 171, 29-37.
32. Zhang KF, Bosch-Serra AD, Boixadera J, Thompson AJ. Investigation of water dynamics and the effect of evapo-transpiration on grain yield of rainfed wheat and barley under a mediterranean environment: a modelling approach. Plos One. 2015; 10.
33. Zhang YY, Hua RX, Xia R. Impact analysis of climate change on water quantity and quality in the Huaihe River Basin. J. Nat. Resour. 2018; 1, 114–126.
34. Zheng J. Study on the utilization of brackish water under the condition of bar cover. Shandong Agricultural University. 2002;
35. Tao J, Tian J, Li J. Study on drip irrigation system of greenhouse pepper under different brackish water film. China Rural Water and Hydropower. 2014; 5, 68-72. (in Chinese).
36. Wang R, Kang Y, Wan S, Hu W, Liu S, Liu S. Salt distribution and the growth of cotton under different drip irrigation regimes in a saline area, Agricultural Water Management. 2011cvol; 100, no. 1, pp. 58–69.
37. Bao SD. Analysis of soil and agrochemistry. Third Edition. Beijing: China Agriculture Press. 2000; [in Chinese].
38. Olsen SR, Cole CV, Watanable FS, Dean L. Estimation of available phosphorus in soils by extraction with sodium bicarbonate. Washington, D.C. United States Department of Agriculture. 1954.
39. Makhdum MI, Pervez H, Ashraf M. Dry matter accumulation and partitioning in cotton (Gossypium hirsutum L.) as in-fluenced by potassium fertilization. Biol Fertil Soils. 2007; 43: 295–301.

Listed responses to comments by the reviewers (PONE-D-22-03631**)**

Dear Prof. Dr. Rafiq Islam

Academic Editor

PLOS ONE,

Many thanks for your letter. We greatly appreciate the constructive suggestions and comments on our MS entitled “Effect of surface water and underground water drip irrigation on Cotton growth and yield under two different irrigation schemes" from both yourself and the reviewers. All those comments are valuable and very helpful for us to improve our manuscript. We extend our great appreciation for taking the time and efforts to provide such insightful guidance. We have taken a complete consideration to all reviewers’ comments as well as those suggestions from the editor’s and have made the corrections one by one in the revised version of our manuscript. The changes in the revised MS are marked in track change model.

We sincerely hope the revised manuscript will be able to meet the requirement and will be finally accepted to publish on your journal of “PLOS ONE”. Of course, we are always available to provide ongoing changes to our manuscript if there is any further request either from you or from the reviews.

Below please find the revised manuscript and the responses. Again, thank you and all the reviewers for your kinder assistance and we are looking forward to hearing from you at your earliest convenience.

Best wishes

Many thanks again

On behalf of the all coauthors

Sincerely yours,

The following is a point-to-point response to the reviewers’ comments.

Comments from the editors and reviewers:

Response to the editors' comments

Editor's note:

When submitting your revision, we need you to address these additional requirements.

Please ensure that your manuscript meets PLOS ONE's style requirements, including those for file naming.

Response: Thank you very much, we have fully consider the PLOS ONE journal style requirements including file naming. Please see our revised manuscript

We note that the grant information you provided in the 'Funding Information' and 'Financial Disclosure' sections do not match.

When you resubmit, please ensure that you provide the correct grant numbers for the awards you received for your study in the 'Funding Information' section.

Response: We are extremely sorry for our careless job. We have now provided the corrected and revised full "Funding Information" in our revised manuscript. For instance

"This work was supported by the Key Technologies Research and Development Program of China (2017YFC0504302-02)".

Please see our revised funding Information section in our revised manuscript.

We note that you have stated that you will provide repository information for your data at acceptance. Should your manuscript be accepted for publication, we will hold it until you provide the relevant accession numbers or DOIs necessary to access your data. If you wish to make changes to your Data Availability statement, please describe these changes in your cover letter and we will update your Data Availability statement to reflect the information you provide.

Response: Thank you very much, Actually we don't have any repository data information, and we don't want to provide any repository information, so therefore please update our data availability statement to no repository data availability statement.

Please include your tables as part of your main manuscript and remove the individual files. Please note that supplementary tables (should remain/ be uploaded) as separate "Supporting Information" files.

Response: Thank you very much. We have included the tables as a part of manuscript. Please check our revised manuscript.

Please remove any funding-related text from the manuscript and let us know how you would like to update your Funding Statement.

Response: Thank you so much for your time. We have removed the funding-related text from the manuscript. Please check our revised manuscript.

Please state what role the funders took in the study. If the funders had no role, please state: "The funders had no role in study design, data collection and analysis, decision to publish, or preparation of the manuscript."

If this statement is not correct you must amend it as needed.

Please include this amended Role of Funder statement in your cover letter; we will change the online submission form on your behalf."

Response: Thank you so very much. "The funders had no role in study design, data collection and analysis, decision to publish, or preparation of the manuscript."

If any authors received a salary from any of your funders, please state which authors and which funders.

Response: Thank you very much for time. "The authors received no specific funding for this work."

The following is a point-to-point response to the reviewers' comments.

Reviewer(s)' Comments to Author:

Reviewer:

<u>Major Issues/Concerns</u>

Response to reviewer # 1: Thank you very much. We appreciate you taking the time to offer us your comments and insights related to the paper. We found your feedback very constructive. We tried to be responsive to your concerns. We hope you find these revisions rise to your expectations.

Regarding Reviewer#1 major concerns, we make an explanation in detail as follow:

1. "Badly unclear written. Revise it as I revised for the first experiment."

<u>Response:</u> Thank you very much for your kind revision. We have revised experiment 2nd, according to the reviewer suggestion. For instance "The first experiment comprised of five ratios of underground water to surface water including; 1:0 (U), 0:1 (S), 1:1 (T_3), 1:2 (T_4) and 1:3 (T_5). While, the second experiment comprised of eight treatments including: 1:3 (T1), 2:2 (T2), 3:1 (T3), 1:3 {S: U (T4)}, 2:2 {S: U (T5)}, 3:1 {S: U (T6)}, 4:0 (T7) and 0:4 (T8)."

Please see our revised manuscript

2. Interactive effect of which factors? You should have at least two factors for an interaction effect.

<u>Response:</u> Many thanks. Basically in this sentence {"Furthermore, two-way ANOVAs revealed a significant main and interactive effect on dry matter accumulation at various growth stages treated with surface water in round irrigation scheme ($p < 0.05$)"} we present the results of two way ANOVA for which after coding the data in experiment 2nd, we consider Surface water (S) as a separate factor and underground water (U) as a separate factor while the S*U is their interactive factor, To be more clear and precise following is the ANOVA tables for Surface water, Underground water and their interaction for various growth stages, but in our manuscript we have linked these ANOVA tables results with their corresponding figures.

Please see figure 3

Table: Results of two-way ANOVAs for growth stages (stem, leaves and roots) of dry matter accumulation as dependent on surface water (*S*), underground water (*U*) and their interaction (S × *U*).

		Stem		Leaves		Roots	
Source	df	F	P-value	F	P-value	F	P-value
S	1	2.36	0.14	3.94	**0.05***	3.06	0.09
U	1	0.73	0.40	0.03	0.95	0.08	0.77
S×U	1	6.23	**0.02***	0.45	0.50	2.20	0.15

Table: Results of two-way ANOVAs for boll stages (stem, leaves and roots) of dry matter accumulation as dependent on surface water (*S*), underground water (*U*) and their interaction (S × *U*).

		Stem		Leaves		Roots	
Source	df	F	P-value	F	P-value	F	P-value
S	1	28.1	**0.00***	12.9	**0.00***	46.1	**0.00***
U	1	3.30	0.08	0.00	0.94	0.31	0.58
S×U	1	297.8	**0.00***	42.7	**0.00***	115.8	**0.00***

3. at various growth stages treated with surface water in round irrigation scheme ($p < 0.05$). Unclear! Revise it.

Response: Done, we have revised the whole sentence. i.e.

"Furthermore, the results of two-way ANOVAs revealed a significant main and interactive effect on dry matter accumulation treated with surface water at cotton plant growth, boll and boll opening stages ($p < 0.05$)."

For instance, the highest leaves dry matter in round irrigation at various growth

What do you mean?

Response: We apologize for the confusion. Basically in this study we used two different irrigation schemes i.e. (1) Different mixing ratio irrigation (2) Round irrigation. In experiment 1st (different mixing ratio) we mix different ratios of surface water and underground water irrigation and after mixing we apply the irrigation water to the cotton field (Please see the attached pictures of the

mixing ratios scheme), however in Experiment 2nd (round irrigation) we apply the surface water and underground water directly to the cotton field without mixing it by using a water meter reader (attached is the picture), So to make it more understandable to the reader we use the term round irrigation.

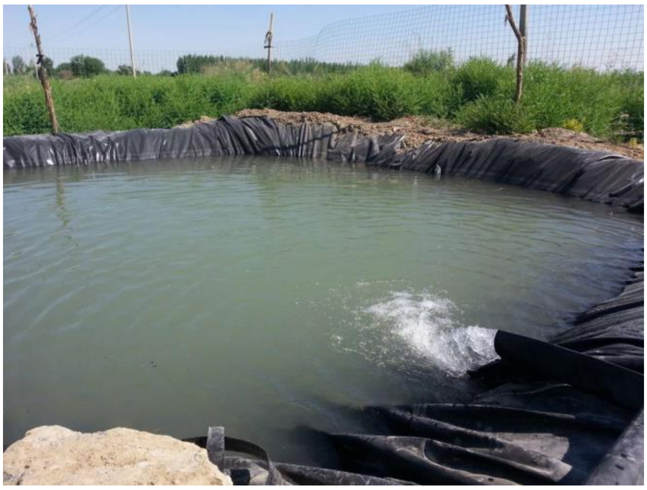

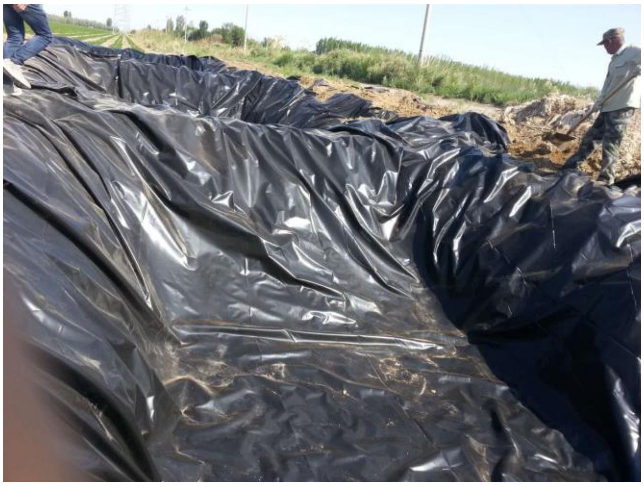

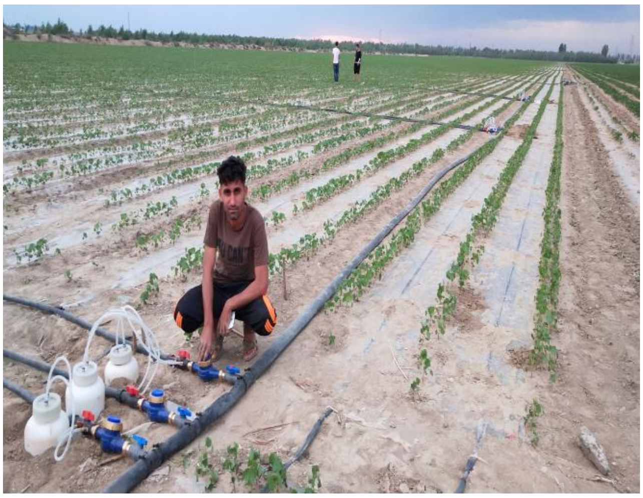

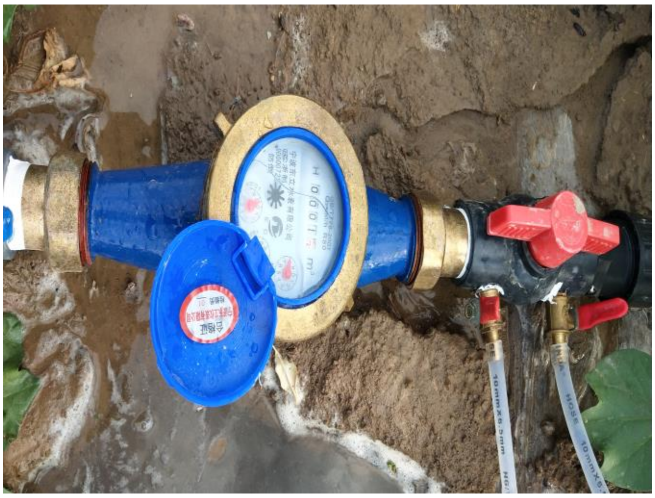

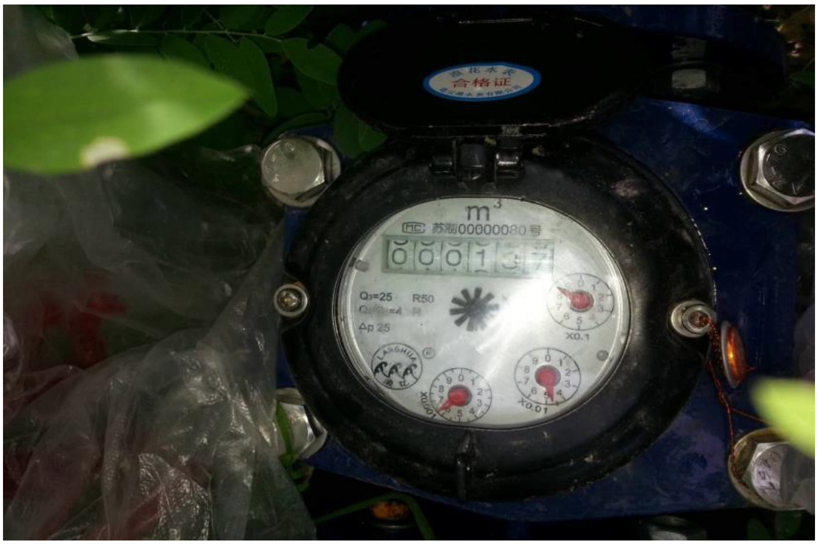

Kg per hectare? Per m2?.

Response: Yes, your consideration is right. The basic unit for cotton is yield in Kg/hm^2, instead of kg/h.m^2, Similar to our studies most of the paper used the same unit following are some of references

Reference:

Suping Wang, Xiaokun Li, Jianwei Lu , Juan Hong, Gang Chen , Xinxin Xue , Jifu Li , Yunxia Wei , Jialong Zou , Guangwen Liu. 2013. Effects of controlled-release urea application on the growth, yield and nitrogen recovery efficiency of cotton Agricultural Sciences 4 (2013) 33-38.

Zhongna Yang, Tarleton State University, Stephenville, TX Mark Yu Ashley Lovell. 2018. COTTON PRODUCTION IN XINJIANG PROVINCE AND ITS IMPACT TO COTTON MARKETS IN CHINA. 2018 Beltwide Cotton Conferences, San Antonio, TX, January 3-5, 2018.

Wang, G.; Feng, L.; Liu, L.; Zhang, Y.; Li, A.; Wang, Z.; Han, Y.; Li, Y.; Li, C.; Dong, H. Early Relay Intercropping of Short-Season Cotton Increases Lint Yield and Earliness by Improving the Yield Components and Boll Distribution under Wheat-Cotton Double Cropping. Agriculture 2021, 11, 1294

This is not a novel and strong conclusion, as your statement looks like a final result of a local report.

Response: Thank you for this direction, we have revised our conclusion in our revised manuscript. Following is the revised conclusion

"Overall, our findings provide useful information to current irrigation practices in water scarce regions. Alternatively, improving water use efficiency is a viable solution to the water scarcity. Therefore, surface water irrigation is recommended as an effective irrigation strategies to improve cotton yield and growth."

Please see our revised manuscript with track changes

Patchy paragraphs.

You should write your introduction and all parts of your manuscript in a linked manner

Response: Thank you very much, we have potentially revised our introduction along with whole manuscript according to the reviewer suggestion. We hope our revised manuscript will meet the reviewer expectation. We strongly focus on paragraph linking and try to remove the patchy words

as well as patchy paragraph.

Please see our revised manuscript

Most parts of your introduction talk about China and its problems, so it can not be interesting for audiences worldwide. It`s a locally issue!

Response: Totally agree. But in china the arid and semi-arid region (52 % land area) faces extreme shortage of irrigation water, especially in Xinjiang. As we have conducted our research study in Xinjiang therefore mostly we talk about Xinjaing water resources and cotton output. However in our introduction part we use some other countries references.

2.2 Soil and Plants sampling

“Move before 2.4”.

Response: Done. Please see our revised manuscript

“If RCBD is your mean!

RCBD: Randomized complete block design

CRD: Completely randomized design.

Be careful in writing the special phrase

Response: Extremely sorry for our careless job. Yes both of the experiment were Randomized complete block design (RCBD), we have carefully revised our both experimental design section.

Please see our revised track change manuscript.

How did you apply your treatments?

More details are needed on irrigation regimes.

Full detail should be presented

Response: Thank you very much. We applied our treatment by digging whole and supply underground water and surface water with a constant ratio by using a water ratio measuring

instrument (please see the attached pictures of our experimental scheme), after mixing surface and underground water we mix the nutrients with the water and supply it to the cotton field through drip irrigation. We have added full information about irrigation regimes please see our revised manuscript.

Please see the attached pictures,

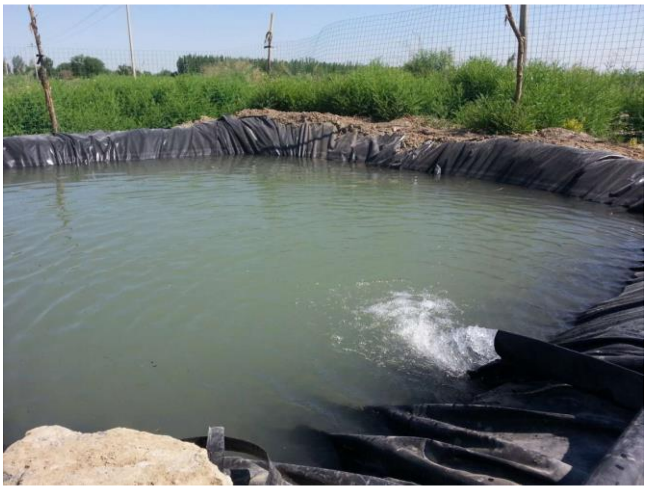

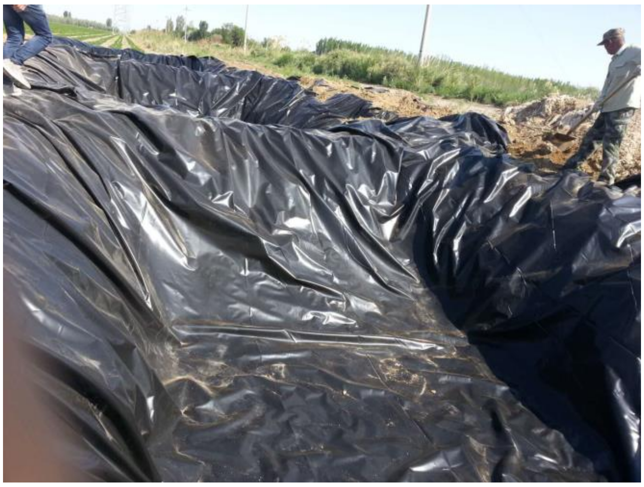

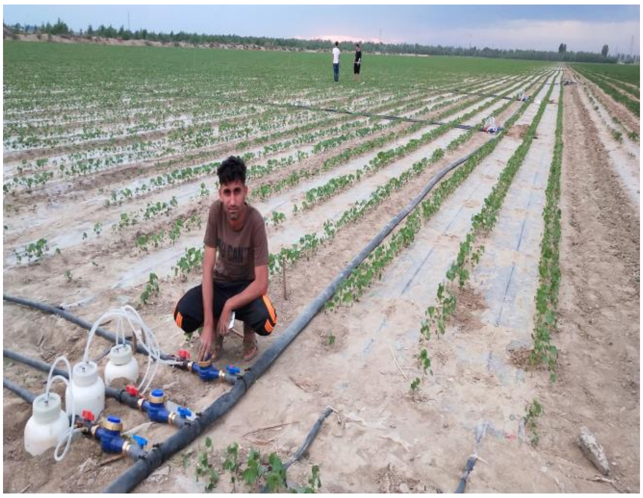

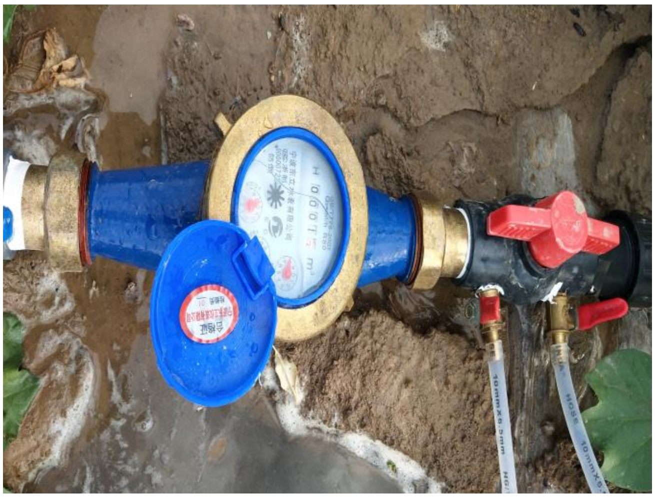

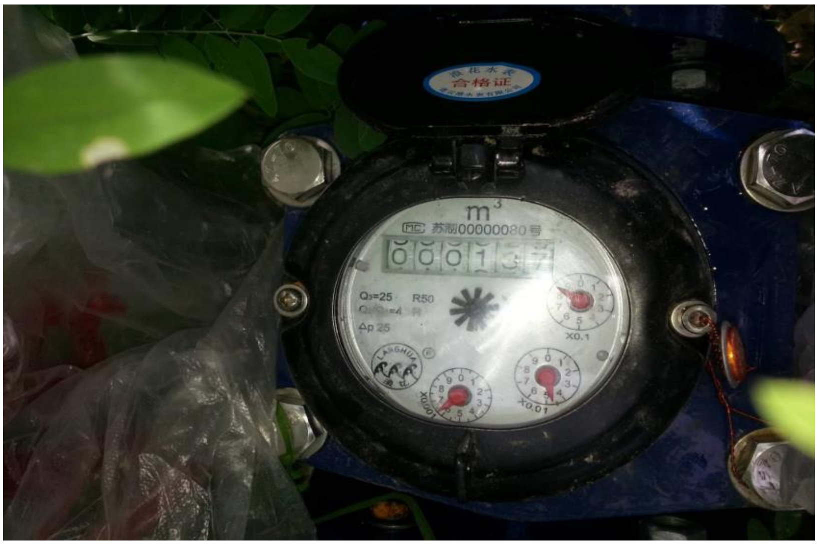

Revise them in a clear way.

Response: Thank you very much for this insightful comment. We have revised our experimental treatments. Please see our revised manuscript.

"The second experiment (round experiment) was a field randomized complete block design (RCBD), comprised of eight treatments including, 1:3 (T_1), 2:2 (T_2), 3:1 (T_3), 1:3 {S:U (T_4)}, 2:2 {S:U (T_5)}, 3:1 {S:U (T_6)}, 4:0 (T_7) and 0:4 (T_8)."

Which factors? You have just one factor!.

Response: We apologize for our mistake. We have corrected it in our revised manuscript. Please see our revised manuscript.

Why didn`t you talk about this in the materials and methods section! This is unclear for us! What do you mean?

Response: We are extremely sorry for the confusion. Now we have included the detailed information about round irrigation in our material and method section. Please see our revised experiment design section in our revised manuscript.

Which methods? You had just one method based on what we saw in the materials and methods section. Your treatments are not different irrigation methods

Response: Thank you very much. Here we talk about our two experimental schemes i.e. 1) Different mixing ratio experiment; 2) Round irrigation experiment. In the first experiment we use different mixing ratio by digging whole and supply constant ratio of surface and underground water into it. After that we mix the nutrients with the water and supply it to the cotton field. However in round irrigation we directly supply the required ratio of surface water and underground water into the cotton field without mixing it.

Just glance at your findings, then discuss them! You should **discuss your results**!

Response: Many thanks. We have completely revised our discussion section, please see our revised manuscript.

This could be a separate chapter without any relationship with your results. In all parts of discussion we should see traces of your work and your results.

Response: We have completely revised our discussion section please see our revised version of manuscript.

Please pay attention to what I said at the end of abstract.

Your conclusion is a bit better than abstract, but not satisfying.

Response: We apologize for the confusion. We have revised our conclusion section in our revised manuscript.

Update the references.

No 2022 article

Just one 2021 reference, which not an article.

Response: Done we have added some new references in our revised manuscript.
