# Supplementary material for: Effect of surface water and underground water drip irrigation on cotton growth and yield under two different irrigation schemes
Source: PLoS One. 2022 Oct 14;17(10):e0274574. doi: 10.1371/journal.pone.0274574 (PMC9565416; doi:10.1371/journal.pone.0274574)
Supplement: Attachment — Submitted filename: ManuscriptRIrEvized.doc [file pone.0274574.s005.doc]

Type of contribution: Research Article

Number of text pages: 17

Number of tables: 8

Number of figures: 6

Title:

Effect of surface water and underground water drip irrigation on cotton growth and yield under two different irrigation schemes

**Nihal Niaz^1^ and Cheng Tang^1^***

^1^College of Agronomy, Shihezi University, Shihezi City, 832003, Xinjiang Province, China. *****Correspondence: [tangcheng1983@163.com](mailto:tangcheng1983@163.com); Tel.: +86 15352608029

Both authors contributed equally to this work

**ABSTRACT:** Two field experiments were conducted to investigate the effects of surface water and underground water drip irrigation on cotton yield, dry matter accumulation, and nutrients uptake under different irrigation regimes,. The first experiment comprised of five ratios of underground water to surface water including 1:0 (U), 0:1 (S), 1:1 (T_3_) 1:4 (T_4_) and 1:3 (T_5_). While, the second experiment included eight treatments: (i) T1 {(1:3) underground water : surface water}; (ii) T2 {U:S (2:2)}; (iii) T3 {U:S (3:1)}; (iv) T4 {S:U (3:1)}; (v) T5 {S:U (2:2)}; (vi) T6 {S:U (1:3)}; (vii) T7 {U:S (4:0)}; and (viii) T8 {U:S (0:4)}. The average concentration of leaves dry matter after 8^th^ irrigation significantly increased by 131.2% (S), 34.4% (U: S= 1:1), 59.3% (U: S= 1:2), and 93.7% (U: S= 1:3), respectively, compared with U treatment. Likewise, the stem dry matter increased from 48.5 g (U), to 122.2 g (S) and 101.6 g (U:S= 1:3). Furthermore, two-way ANOVAs revealed a significant main and interactive effect on dry matter accumulation at various growth stages treated with surface water in round irrigation scheme (p < 0.05). For instance, the highest leaves dry matter in round irrigation at various growth stages were recorded in T8 treatment. Compared with T7 treatment an average increase rate of 50.6 % (growth), 100.9 % (boll) and 93.3 % (boll opening), in stem dry matter were recorded in T8 treatment. The soil available N at 0-20 cm after 8^th^ irrigation recorded an average increase rate of 40.1%, 6.6%, 13.5%, and 29.5%, respectively, while at 20-40cm an average increase rate of 37.4 %, 7.1%, 20.0%, and 21.9% were noted (p < 0.05). Moreover, the concentration of N in round irrigation at 0–20 cm at different growth stages were 83.3±2.8 (growth stage), 79.01±1.84 (boll stage), 96.16±3.83 (boll opening stage) in T8, while in T7 the concentration of N was 36.1±5.9 (growth), 54.51±2.81 (boll), and 53.9±3.83 (boll opening) (p < 0.05). The highest cotton yield of 6571 kg/hm^2^ was observed in S treatment compared with the U treatment (5492 kg/hm^2^, U: S= 1:1 (5502 kg/hm^2^), U: S= 1:2 (5873 kg/hm^2^) and U: S= 1:3 (6111 kg/hm^2^). Similarly, in round irrigation the increasing trend follow the order of T8 > T1 > T4 > T2 > T5 > T3 > T6 > T7. Therefore, it is suggested that surface water irrigation should be applied to maximize the cotton yield, dry matter accumulation, nutritional status and overall growth of cotton crop.

**Keywords:**;;; dry matter accumulation; N,P,K uptake;; underground water.

**1. Introduction**

Irrigation water is one of the most limiting factors for future global agricultural development [1, 2]. Arid and semi-arid regions of the world are being subjected to severe water shortages due to climate change and over-exploitation of water resources [3]. Taken China is an example, arid and semi-arid area occupies 52.5% of China’s land area which is mainly distributed in Northwest, Northeast, and North China [4]. Agriculture sectors in these regions are totally dependent on sufficient irrigation. To fulfill the inherent irrigation water requirements, about 60% of the water resources including both surface and underground water is used for agriculture purposes. On contrast, due to significant increase in population, there is competition for fresh water among municipal, industrial and agricultural sectors in several countries in the world especially in China [5]. Consequently, the allocation of fresh water to agriculture sector in these regions substantially decreased [6]. Therefore, improving irrigation efficiency is the key and effective way to solve the problem of water shortage, ecological environment deterioration, and to develop agricultural production in arid and semi-arid regions.

Cotton (*Gossypium* *hirsutum* L.), an important source of natural fibers to textile industries that serve the humanity from at least more than four to seven thousand years ago [7]. The current global cotton fiber production is estimated to be 24.65 million tons, with 6.71 million tons produced in America, 0.38 million tons produced in Europe, and 15.06 million tons produced in Asia [8]. Following this, China accounts for one-quarter of the world’s cotton output and one-third of the world’s cotton consumption [9]. However, cotton production is completely dependent on irrigation i.e., the shortage of irrigation water resources restricts the comprehensive improvement of cotton productivity [10, 11]. Water requirements for cotton growth vary from 700 to 1200 mm during the growing season, depending on irrigation method, and production goals [12]. Numerous studies have been conducted to examine water-use efficiency of cotton in recent years. For instance, a study conducted by Grismer [13] found that, in Arizona counties, for upland cotton actual evapotranspiration (ETc) water-use efficiency varied from 1.27 to 1.38 kg/ha-mm while, for pima cotton, it varied from 0.9 to 1.09 kg/ha-mm. In California counties, ETc water-use efficiency varied from 1.34 to 2.10 kg/ha-mm and 1.51–1.77 kg/ha-mm for upland and pima varieties, respectively. Both surface water resources (water from rivers and reservoirs) and groundwater resources (water stored in aquifers) were used for agriculture purposes in arid and semi-arid regions. However, irrigation with underground water has a negative impact on cotton productivity, plant nutritional condition, and dry matter accumulation. For instance, excessive underground water irrigation exacerbates the soil salinization problems, and reduce crop yield [11, 14]. Likewise, well water irrigation with low temperature potentially inhibits the growth and development of cotton plant [15]. Moreover, low soil temperatures may slow down the uptake rate of nutrients such as N so much that they turn out to limit vegetative growth rates [16]. At 20°C-RZT, nutrient concentrations have been significantly affected plant growth indexes, indicating that low root temperature inhibited high nutrient effects on plant growth [16, 17]. While on the other hand, surface water irrigation shows promising effect on increasing crops yield [18, 19]. For example, irrigating seedlings with warm water (surface water) can increase the stem thickness, leaf area, root coefficient, photosynthetic rate, dry matter per unit fresh weight, root-shoot ratio, and strong seedling index of seedlings [16]. Therefore, clarifying the effect of surface water and underground water on cotton growth and yield by applying different irrigation regime is of paramount importance for improving crop productivity by using less water.

Xinjiang Uygur Autonomous Region is one of the most water-scarce states in China. According to the Statistics Bureau of Xinjiang Uygur Autonomous Region, the water production per unit area of Xinjiang Uygur Autonomous Region is 51 mm, which is the second highest rank in the country [19]. The large number of glaciers in Xinjiang Uygur Autonomous Region and the small unit area are important issues of water resources in Xinjiang Uygur Autonomous Region. Snowmelt water from glaciers accounts for more than 25% of total surface water [20]. However, according to China’s statistics, Xinjiang's total cotton output in 2017 was 4.082 million tons, accounting for 74.4% of the country's total production [20]. While on the other side, agriculture in Xinjiang is totally dependent on irrigation, according to 2016 Xinjiang Water Resource Bulletin, agriculture consumes 94.3% of the available total water resources.

In the present-day context, lot of emphasis is being given on improvements in irrigation practices to increase crop production and to sustain the productivity levels. In this study, two field experiments were carried out in calcareous soils. We hypothesize that surface water drip irrigation outcompetes underground water irrigation in increasing cotton yield, dry matter accumulation and NPK uptake. Therefore, the objectives of our study were to (i) compare the effects of different irrigation schemes on cotton growth and yield. (ii) Clarify the response of cotton growth to surface and underground water addition, (iii) finally put forward an appropriate irrigation strategy to increase cotton yield in calcareous soil.

**2. Materials and Methods**

**2.1 Experimental site**

The study area is located in the Mosuowan reclamation region (44°03′N, 86°05′E), which is located on the northern slope of Tianshan Mountain in Xinjiang and is surrounded by the Gurbantunggut Desert. According to WRB soil taxonomy, the tested soil is classified as Calcisol Fluvisols. The study area has a typical continental climate with a mean annual precipitation of 115 mm, and rainfall largely occurs from April to July. The mean annual potential evapotranspiration is approximately 2,000 mm. The physicochemical characteristics of given soil is listed in (Table 1).

**Insert “(Table 1)”**

**2.2 Soil and Plants sampling**

Soil samples were collected at depths of 0-20 cm and 20-40 cm from each block after 3-5 days of irrigation. Samples were air dried, sieved through 1mm and 0.15 mm for nutrients determination. The pH was determined at soil to milli-Q water ratio of 1:5 w/v using pH meter. The organic matter was determined by potassium dichromate volumetric method. Nitrogen was determined by semimicro-Kjeldahl method [37]. Phosphorus was measured by the perchloric acid digestion method [38]. Potassium was determined by flame photometry [37]. Soil available Nitrogen, soil available phosphorus, soil available potassium and soil organic matter were determined in soil samples after growth stage, boll stage and boll opening stage.

Plant samples were randomly collected from each block at interval of 3-5 days after each irrigation. Plant samples were divided into the following parts (leaves, stems, roots and fruits), washed with tap water and then dried in oven at 105˚C for 30 minutes and then at 75˚C for 3 days. The plant samples were then weighed with balance and the dry matter data were calculated after growth stage, boll stage and boll opening stage, following the method of [39] (Figure 1).

**Insert “(Figure 1)”**

**2.3 Experimental Design**

The first experiment was a randomized complete block design field experiment done on April 12, 2019 with five treatments including U (underground water); S (surface water); U:S= 1:1; U:S= 1:2; U:S= 1:3, respectively. Each treatment was replicated three times while the area of each block was 55.2 m^2^. The supply of water and nutrients for each irrigation has listed in (Table 2).

**Insert “(Table 2)”**

The second experiment was a field randomized complete block design (RCBD) having eight treatments consisted of U:S=1:3, U:S =2:2, (iii) T3 {U:S (3:1)}; (iv) T4 {S:U (3:1)}; (v) T5 {S:U (2:2)}; (vi) T6 {S:U (1:3)}; (vii) T7 {U:S (4:0)}; and (viii) T8 {U:S (0:4)}. The treatment ratios represents the supply of surface and underground water in different stages of cotton growth i.e., seedling stage, growth stage, boll stage and boll opening stage. Further details about irrigation is given in (Table 3). Nutrients (fertilizer) and water were supplied through drip irrigation. The supply of water and nutrients for each irrigation has listed in (Table 4).

**Insert “(Table 3 and 4)”**

**2.4 Statistical analysis**

Data were analyzed using the SPSS 25.5 statistical program (SPSS Inc., Chicago, IL, USA) with two-way ANOVA at a significance level of p < 0.05. A Duncan multiple range test was carried out to test the significant differences between different treatments. GraphPad Prism 12.0 software (GraphPad Software, Inc., San Diego, CA, USA) was used for data processing and images making. All results in figures and tables were presented as mean ± standard deviation (SD) of three replicates, and a significance level of P <0.05 was used for all analysis.

**3. Results**

**3.1 Effects of different mixing ratio irrigation method on the dry matter accumulation**

The average dry matter accumulation in the different parts of the cotton plant fluctuated greatly with different irrigation treatments and increased sharply after each irrigation time (Figure. 2). For instance, the significant maximum dry weights (DW) were recorded in S treatment followed by the U: S= 1:3 treatment. However, the lowest dry matter accumulation was recorded in the U treatment. The concentration of leaves dry matter after 8^th^ irrigation increased from 32.8 ± 0.15 g (U), to 74.1 ± 0.27g (S), 43.5 ± 0.18g (U:S= 1:1), 51.1 ± 0.21g (U:S= 1:2) and 62.9 ± 0.37g (U:S= 1:3), with an average increase rate of 131.2%, 34.4%, 59.3%, and 93.7%, respectively (p < 0.05, Figure 2a). Compared with underground water the tremendous average increase rate of 131.2% and 93.7% were noted in surface water and different mixing ratios. Similarly, stem dry matter of five applied treatments as affected by eight irrigation regimes are presented in Figure 2b. The concentration of stem dry matter after 8^th^ irrigation increased from 48.5 g (U), to 122.2 g (S) and 101.6 g (U:S= 1:3), with an average increase rate of 151.5 % and 109.5%, (Figure 2b). Moreover, compared with underground water irrigation treatment (24.6 g), a significant highest root dry matter was recorded with surface water treatment (51.74 g) and U: S= 1:3 treatment (46.96 g) (p < 0.05, Figure 2c). Similarly, compared with surface water, underground water significantly suppressed roots dry matter accumulation and follows the sequence of S> U: S= 1:3> U: S= 1:2> U: S= 1:1>U. The highest fruits dry matter accumulation was recorded in the treatment of surface water (83.43 ± 0.34 g) compared with all other treatments (p < 0.05, Figure 2d).

**Insert “(Figure 2)”**

**3.2 Effects of round irrigation method on dry matter accumulation**

Round irrigation method also significantly affected dry matter accumulation in the different parts of the plant at various stages of growth (p < 0.05, Figure. 3). The biomass of yield-related organs showed a trend of gradual increase with the progression of the growth period, and the most intense change was at the beginning of the boll development stage. Maximum dry weights (DW) were achieved at boll opening stage and minimum at growth stage. The DW of leaves, stem, roots and fruits continued to increase initially and then declined and stayed stable comparatively because of leaf senescence and termination of reproductive development at final stages (Figure 3a-d). The highest leaves dry matter of 14.4g (growth stage), 88.8g (boll stage) and 100.7g (boll opening stage) were observed in T8 treatment compared with the lowest, 8.9g (growth stage), 42.9g (boll stage) and 78.7g (boll opening stage) of T7 treatment. Results of Two-way ANOVAs reveals a significant main and interactive effect on various stage of leaves dry matter treated with surface water (p < 0.05, Figure. 3a). Compared with T7 treatment an average increase rate of 50.6 %, 100.9 % and 93.3 %, at growth, boll and boll opening stage respectively in stem dry matter were noted in T8 treatment. Likewise, a significant main and interactive effect were observed at various stages of stem dry matter (p < 0.05, Figure. 3b). Also, it was evident from the results that the highest root dry matter recorded in growth, boll and boll opening stage were 6.46g, 38.68g and 51.63g with T8 treatment, compared with the lowest values of 4.60g, 12.24g and 23.57g recorded in T7. A significant main and interactive effect were observed at boll and boll opening stages of roots dry matter (p < 0.05, Figure. 3c). Fruits dry matter were produced in boll and boll opening stages and was substantially higher in T8 treatment compared with other applied treatments throughout the whole experiment. A significant main interaction effect on boll and boll opening stage while a significant interactive effect on boll stage were observed for fruits dry matter (p < 0.05, Figure. 3d). Our result showed that the surface water irrigation along with mixing ratio shows promising effect on average dry matter accumulation in cotton plant.

**Insert “(Figure 3)”**

**3.3 Effects of different mixing ratio and round irrigation on soil available nutrients**

Soil available nitrogen at a depth of 0–20 and 20–40 cm fluctuated greatly during the whole cotton growth period, and increased sharply after each irrigation time (Figure. 4a, b). The concentration of N at 0–20 cm after 8^th^ irrigation increased from 72.3 ± 0.15 mg/kg (U), to 101.3 ± 0.27 mg/kg (S), 77.1 ± 0.18 mg/kg (U:S=1:1), 82.1 ± 0.21 mg/kg (U:S=1:2) and 99.3 ± 0.37 mg/kg (U:S=1:3), with an average increase rate of 40.1%, 6.6%, 13.5%, and 29.5%, respectively (p < 0.05, Figure 4a). Compared with U treatment an average increase in N concentration at 20–40 cm after 8^th^ irrigation in S treatment were 37.4 %, 7.1%, 20.0%, and 21.9% (p < 0.05, Figure 4b). Trends of soil available nitrogen follows the order of S> U:S=1:3> U:S=1:2> U:S=1:1>U. Moreover, the accumulation of P in different soil depths 0–20 and 20–40 cm was presented in (Figure. 4c, d). The concentration of P at 0–20 cm after 8^th^ irrigation increased by 76.9%, and 33.8% %, respectively in S and U:S=1:3 treatments compared with U treatment (p < 0.05, Figure 4c). Whilst at 20-40 cm, the amount of P in surface water application significantly increased from 12.6 ± 0.15 mg/kg (U), to 28.4 ± 0.27 mg/kg (S), 13.4 ± 0.18 mg/kg (U:S=1:1), 13.3 ± 0.21 mg/kg (U:S=1:2) and 22.6 ± 0.37 mg/kg U:S=1:3 (p < 0.05, Figure 4d). The status of available potassium in soil at 0–20 and 20–40 cm remains parallel throughout the experiment (Figure 5a, b). Compared with other treatments the surface water treatment shows maximum concentration of potash, however the difference between different applied treatments were negligible. Soil organic matter was significantly affected by different irrigation treatments (Fig. 5c, d). However, at the start of the experiment the S treatment followed by U: S=1:3 treatment shows potential promising effect at both 0–20 and 20–40 cm, while the difference between other treatments were negligible.

Furthermore, the concentration of soil available nutrients at a depth of 0-20cm and 20-40 cm in round irrigation method significantly affected by different applied treatments. The concentration of N recorded at 0–20 cm at different growth stages were 83.3±2.8 (growth stage), 79.01±1.84 (boll stage), 96.16±3.83 (boll opening stage) in T8, while in T7 the concentration of N was 36.1±5.9 (growth), 54.51±2.81 (boll), and 53.9±3.83 (boll opening) (p < 0.05, Table 5). The soil available P content significantly increased in the initial and final stages with T8 treatment throughout the experiment (p < 0.05, Table 6). While, the status of available potassium in soil remains parallel throughout the experiment, the difference between different applied treatments were negligible (p < 0.05, Table 7). However, the maximum concentration of potash was determined in T8 treatment. Soil organic matter was significantly affected by different ir-rigation treatments (p < 0.05, Table 8). However, at the start of the experiment the surface water irrigation treatment shows promising effect at both 0–20 and 20–40 cm, while the difference between other treatments were negligible.

**Insert “(Figure 4 and Figure 5)”**

**Insert “(Table 5, Table 6, Table 7 and Table 8)”**

**3.4 Effects of different irrigation methods on cotton yield**

The effect of different mixing ratio irrigation on cotton yield is presented in Figure 6a. Cotton yield responded significantly to different water irrigation modes. For instance, the maximum cotton yield 6571 kg/hm^2^ was observed in (S) treatment compared with the treatment (U) (5492 kg/hm^2^), U: S= 1:1 treatment (5502 kg/hm^2^), U: S= 1:2 treatment (5873 kg/hm^2^) and U: S= 1:3 treatment (6111 kg/hm^2^). Likewise, round irrigation potentially effect cotton yield under different applied treatments (Figure 6b). For example, the increasing trend follow the order of T8 > T1 > T4 > T2 > T5 > T3 > T6> T7, which shows that surface water irrigation can effectively guarantee cotton production. The highest cotton yield was observed in T8 treatment in which surface water was supplied through all stages of cotton growth, followed by treatment T1 and T4 (Figure 6b). However, the lowest yield was recorded in treatment T7 in which under-ground water was supplied.

**Insert “(Figure 6)”**

**4. Discussion**

Water for irrigation is a major limitation to agricultural production in the Xinjiang region. The Xinjiang region in Northwest China is one of the most important cotton producers. The cotton plantation area in Xinjiang is 1.8 × 106 ha, accounting for 54% of China’s total cotton planting area [20]. It produced 451 × 104 tons of cotton ( in 2014, accounting for 73% of China’s total cotton production [18]. Meanwhile, surface water evaporation caused by high temperatures results in a severe water shortage in southern Xinjiang leading to soil salinization, a lowered survival rate for crops, and slow development of local agriculture [21]. Similarly, increased usage of underground water for irrigation exacerbates the soil salinization problems, which significantly reduce crop yield [10, 11]. In line with the previous findings, in these experiments surface water application in both round and mixing ratio irrigation methods, significantly promotes dry matter accumulation (Fig. 2-3), NPK uptake (Fig. 4,5 and Table 5-8) and cotton yield (Fig. 6), compared with all other applied treatments. The most likely reasons may be, in general surface water possess high temperature while the underground water temperature is quite low. Previous study has shown that well water irrigation with low temperature potentially inhibits the growth and development of jujube [22]. Likewise, several published literatures have shown that underground water irrigation (low temperature water) affect the growth, yield, dry matter accumulation and active developmental stages of grains plants such as peanuts, cucumber, and tomato [23, 24, 25, 26]. For instance, a study conducted by Meng et al. (2016), noted that underground water irrigation significantly affects the growth and development of cotton plant. Deng et al., [27], also pointed out that underground water irrigation along with their low temperature properties significantly retarded the growth of vegetables and their photosynthetic developments. Consequently, the excessive usage of underground water irrigation results in the accumulation of toxic substances in soil which alternatively leads to reduction in plants and grains yields [28, 29]. The accumulation of salt can directly decrease soil nutrient efficiency by inhibiting microbial mineralization activity in soil [30]. Additionally, salinity can also indirectly affect soil nutrient cycling and efficiency by destroying soil physical structure [31, 32, 33]. The study of zhang et al., 2002 [34] showed that alternate irrigation with surface fresh water can reduce soil salt content and increase cotton production. In this study the different mixing ratios also shows promising effects on cotton yield and dry matter accumulation when compared with underground water treatment alone, this is possibly due to when the underground water and surface water were mixed together, the temperature and salt content were changed. For example, a study conducted by Tao et al. 2014 [35], showed that mixed irrigation mode of brackish and fresh water with a salinity of 1.6g/L could achieve higher crop yield with better quality. Consistently, a study carried out by Wang et al. 2010 [36], showed that well and canal mixed irrigation could keep the salt balance of root soil even in relatively dry years, while well irrigation alone results in salt accumulation in roots of winter wheat and decreased the yield up to 20% -30%. All these findings suggest that under-ground water irrigation possess negative effects on plant yield and growth whilst sur-face water irrigation and different mixing ratios significantly promote cotton yield NPK, uptake and dry matter accumulation.

**5. Conclusions**

It can be concluded that surface water along with their different mixing ratios irrigation outcompete underground water irrigation in both applied irrigations methods. The dry mater accumulation, nutrients uptake and cotton yield at various stages i.e., growth stage, boll stage, and boll opening stage were significantly higher in surface water treatments compared with underground water treatment. Overall, our findings provide meaningful information to current irrigation practice in increasing cotton growth and yield. We suggest that surface water irrigation application is recommended as an effective irrigation strategy in Xinjiang calcareous soil for better cotton yield and nutrient uptakes.

**Disclosure statement**

The authors declare that they have no conflict of interest.

**Funding**

This research was jointly supported by National Natural Science Foundation of China (41161047), and the Twelfth Five-Year National Technology Support Program (2012BAD42B02).

**References**

1. UN-Water. The United Nations World Water Development Report 4: Managing Water under Uncertainty and Risk; United Nations World Water Assessment Programme, UNESCO: Paris, France, 2012.
2. Gu Z, Qi Z, Ma L, Gui D, Xu J, Fang Q, Yuan S, Feng G. Development of an irrigation scheduling software based on model predicted crop water stress. Comput. Electron. Agric. 2017; 143, 208–221.
3. Feike T, Ling YK, Mamitimin Y, Nan H, Lin L, Abdusalih N, Xiao H, Doloschutz R. Determinants of cotton farmers’ irrigation water management in arid northwestern china. Agric. Water Manag. 2017; 187, 1–10.
4. Wei Qu, Yanmei Tan, Zhentao Li, Eefje Aarnoudse, Qin Tu. Agricultural Water Use Efficiency—A Case Study of Inland-River Basins in Northwest China. Sustainability. 2020; 12, 10192.
5. Meng B, Liu JL, Bao K, Sun B.Water fluxes of Nenjiang river basin with ecological network analysis: Conflict and coor-dination between agricultural development and wetland restoration. J. Clean. Prod. 2019; 213, 933–943.
6. Liu Y, Jiang H, Li C, Huang H, Pan Z, Chai C. Analysis of irrigation water requirement and irrigation requirement index for cotton of Hebei province. Nongye Gongcheng Xuebao/Trans. Chin. Soc. Agric. Eng. 2013; 29, 98–104.
7. Li Y, Gao X, Tenuta M, Gui D, Li X, Xue W, Zeng F. Enhanced efficiency nitrogen fertilizers were not effective in reducing N2O emissions from a drip-irrigated cotton field in arid region of Northwestern China. Sci. Total Environ. 2020; 748, 141543.
8. FAOSTAT. 2021. Available online: http://www.fao.org/faostat/en/#data/QCL. (accessed on 25 July 2021).
9. Ma C, Mamat S, Yao J, Isak G. Spatio-temporal changes of cotton production in China from 1950 to 2015. Acta Geogr. Sin. 2020; 75, 1699–1710.
10. Sorensen RB, Lamb MC, Butts CL. Crop rotation, irrigation system, and irrigation rate on cotton yield in southwestern Georgia. Crop. Forage Turfgrass Mgmt. 2020; 6, e20053.
11. Mahmoodi-Eshkaftaki M, Rafiee MR. Optimization of irrigation management: a multi-objective approach based on crop yield, growth, evapotranspiration, water use efficiency and soil salinity. Journal of Cleaner Production. 2020; 252, 119901.
12. Evett SR, Baumhardt RL, Howell TA, Ibragimov NM, Hunsaker DJ. Cotton. Crop Yield Response to Water; FAO ir-rigation and drainage paper. No. 66; FAO: Rome, Italy. 2012; pp. 152–161.
13. Grismer ME. Regional cotton lint yield, ETc, and water value in Arizona and California. Agric. Water Manag. 2002; 54, 227–242.
14. Sekhon KS, Kaur A, Thaman S, Sidhu A S, Garg N, Choudhary O P, Buttar G S, Chawla N. Irrigation water quality and mulching effects on tuber yield and soil properties in potato (*Solanum* *tuberosum* L.) under semi-arid conditions of Indian Punjab. Field Crops Research. 2020; 247, 107544.
15. Meng A. Effect of drip irrigation temperature on grey desert soil environment and cotton growth. Urumqi: Xinjiang Agricultural University. 2016; 39-40.
16. Kong Q. Analysis on the key factor of inhibiting cotton production development and discussion on its strategies. Xinjiang Agric. Sci. 2010; 47, 3–5 (in Chinese with English abstract).
17. Kang Yaohu, Wang Ruoshui, Wan Shuqin, Hu Wei, Jiang Shufang, Liu Shiping. Effects of different water levels on cotton growth and water use through drip irrigation in an arid region with saline ground water of Northwest China. Agric. Water Manag. 2013; 109, 117– 126.
18. Chen Z, Niua Y, Zhaoa R, Hana C, Hanb H, Luoa H. The combination of limited irrigation and high plant density optimizes canopy structure and improves the water use efficiency of cotton. Agric. Water Manag. 2019; 218, 139–148.
19. Wang J, Zhao S, Tan X, Liang Y, Gong Z, Ai X, Guo J, Maimaiti M, Li X, Zheng J. Production situation and cotton seed industry development report in Xinjiang cotton planting region in 2018. Cotton Sci. 2019; 41, 9–14.
20. Tian JS, Zhang XY, Yang YL, Yang CX, Xu SZ, Zuo WQ, Zhang WF, Dong HY, Jiu X L, Yu Y C, Zhao Z. How to reduce cotton fiber damage in the Xinjiang China. Industrial Crops and Products. 2017; 109, 803-811.
21. Fang S, Tu W, Mu L, Sun Z, Yang Y. Saline alkali water desalination project in southern Xinjiang of China: a review of desalination planning, desalination schemes and economic analysis. Renewable and Sustainable Energy Reviews. 2019; 113, 109268.
22. Zhang RW, Tian JC, Ma JM. The effect of different irrigation water temperatures on the growth and photosynthesis of leaf lettuce．China Rural Water and Hydropower. 2017; 4, 1－2.
23. Bai YY, Wang HD, Li M. et al. Effect on different irrigation water temperatures on tomato seedlings growth in greenhouse. Water Saving Irrigation. 2012; 11, 16－17.
24. Shi PX, Liu YR, Zhang XJ. et al. Effects of irrigation with low temperature water on soil enzyme activity and soil nutrient of peanut rhizosphere. Chinese Journal of Oil Crop Sciences. 2016; 38, 811－816.
25. Kuang W, Gao X, Gui D, Tenuta M, Flaten DN, Yin M, Zeng FJ. Effects of fertilizer and irrigation management on nitrous oxide emission from cotton fields in an extremely arid region of northwestern China. Field Crop Research. 2018; 229, 17-26.
26. Sun SJ, Cui SM, Song Y. et al. Effects of root zone temperature on growth and photosynthetic parameters of grafted cu-cumber. Agricultural Biotechnology. 2018; 7, 27－31.
27. Deng HL, Tian JC, Ouyang Z. Effect of Irrigation water temperature on soil temperature in vegetable root zone in greenhouse. Ningxia Engineering Technology. 2018; 17, 97－101.
28. Bumgaer NR, Scheerens JC, Mullen RW, et al. Root-zone temperature and nitrogen affect the yield and secondary me-tabolite concentration of fall and spring grown, high-density leaf lettuce. Journal of the Science of Food ＆ Agriculture. 2012; 92, 116－124.
29. Li LJ, Lu XC, Ma HY. et al. Comparative proteomic analysis reveals the roots response to low root-zone temperature in Malus baccata. Journal of Plant Research. 2018; 131, 865－878.
30. Rath KM, Rousk J. Salt effects on the soil microbial decomposer community and their role in organic carbon cycling: a review. Soil Biology Biochemistry. 2015; 81, 108-123.
31. Lakhdar A, Rabhi M, Ghnaya T, Montemurro F, Jedidi N, Abdelly C. Effectiveness of compost use in salt-affected soil. Journal of Hazardous Materials. 2009; 171, 29-37.
32. Zhang KF, Bosch-Serra AD, Boixadera J, Thompson AJ. Investigation of water dynamics and the effect of evapo-transpiration on grain yield of rainfed wheat and barley under a mediterranean environment: a modelling approach. Plos One. 2015; 10.
33. Zhang YY, Hua RX, Xia R. Impact analysis of climate change on water quantity and quality in the Huaihe River Basin. J. Nat. Resour. 2018; 1, 114–126.
34. Zheng J. Study on the utilization of brackish water under the condition of bar cover. Shandong Agricultural University. 2002;
35. Tao J, Tian J, Li J. Study on drip irrigation system of greenhouse pepper under different brackish water film. China Rural Water and Hydropower. 2014; 5, 68-72. (in Chinese).
36. Wang R, Kang Y, Wan S, Hu W, Liu S, Liu S. Salt distribution and the growth of cotton under different drip irrigation regimes in a saline area, Agricultural Water Management. 2011cvol; 100, no. 1, pp. 58–69.
37. Bao SD. Analysis of soil and agrochemistry. Third Edition. Beijing: China Agriculture Press. 2000; [in Chinese].
38. Olsen SR, Cole CV, Watanable FS, Dean L. Estimation of available phosphorus in soils by extraction with sodium bicarbonate. Washington, D.C. United States Department of Agriculture. 1954.
39. Makhdum MI, Pervez H, Ashraf M. Dry matter accumulation and partitioning in cotton (Gossypium hirsutum L.) as in-fluenced by potassium fertilization. Biol Fertil Soils. 2007; 43: 295–301.
