# Supplementary material for: Effect of surface water and underground water drip irrigation on cotton growth and yield under two different irrigation schemes
Source: PLoS One. 2022 Oct 14;17(10):e0274574. doi: 10.1371/journal.pone.0274574 (PMC9565416; doi:10.1371/journal.pone.0274574)
Supplement: Attachment — Submitted filename: Response to Reviewers.doc [file pone.0274574.s006.doc]

**Listed responses to comments by the reviewers (**PONE-D-22-03631**)**

Dear Prof. Dr. Rafiq Islam
Academic Editor

PLOS ONE,

Many thanks for your letter. We greatly appreciate the constructive suggestions and comments on our MS entitled “Effect of surface water and underground water drip irrigation on Cotton growth and yield under two different irrigation schemes" from both yourself and the reviewers. All those comments are valuable and very helpful for us to improve our manuscript. We extend our great appreciation for taking the time and efforts to provide such insightful guidance. We have taken a complete consideration to all reviewers’ comments as well as those suggestions from the editor’s and have made the corrections one by one in the revised version of our manuscript. The changes in the revised MS are marked in track change model.

We sincerely hope the revised manuscript will be able to meet the requirement and will be finally accepted to publish on your journal of “PLOS ONE”. Of course, we are always available to provide ongoing changes to our manuscript if there is any further request either from you or from the reviews.

Below please find the revised manuscript and the responses. Again, thank you and all the reviewers for your kinder assistance and we are looking forward to hearing from you at your earliest convenience.

Best wishes

Many thanks again

On behalf of the all coauthors

Sincerely yours,

-------------------------------------------------------------------------------------------------------

The following is a point-to-point response to the reviewers’ comments.

**Comments from the editors and reviewers:**

**Response to the editors' comments**
Editor's note: 

When submitting your revision, we need you to address these additional requirements.

Please ensure that your manuscript meets PLOS ONE's style requirements, including those for file naming. 

**Response:** Thank you very much, we have fully consider the PLOS ONE journal style requirements including file naming. Please see our revised manuscript

We note that the grant information you provided in the ‘Funding Information’ and ‘Financial Disclosure’ sections do not match.

When you resubmit, please ensure that you provide the correct grant numbers for the awards you received for your study in the ‘Funding Information’ section. 
**Response:** We are extremely sorry for our careless job. We have now provided the corrected and revised full “Funding Information” in our revised manuscript. For instance

“This work was supported by the Key Technologies Research and Development Program of China (2017YFC0504302-02)”.

Please see our revised funding Information section in our revised manuscript.

We note that you have stated that you will provide repository information for your data at acceptance. Should your manuscript be accepted for publication, we will hold it until you provide the relevant accession numbers or DOIs necessary to access your data. If you wish to make changes to your Data Availability statement, please describe these changes in your cover letter and we will update your Data Availability statement to reflect the information you provide.

**Response:** Thank you very much, Actually we don’t have any repository data information, and we don’t want to provide any repository information, so therefore please update our data availability statement to no repository data availability statement.

Please include your tables as part of your main manuscript and remove the individual files. Please note that supplementary tables (should remain/ be uploaded) as separate "Supporting Information" files.

**Response:** Thank you very much. We have included the tables as a part of manuscript. Please check our revised manuscript.

Please remove any funding-related text from the manuscript and let us know how you would like to update your Funding Statement.

**Response:** Thank you so much for your time. We have removed the funding-related text from the manuscript. Please check our revised manuscript.

Please state what role the funders took in the study. If the funders had no role, please state: "The funders had no role in study design, data collection and analysis, decision to publish, or preparation of the manuscript."

If this statement is not correct you must amend it as needed.

Please include this amended Role of Funder statement in your cover letter; we will change the online submission form on your behalf."

**Response:** Thank you so very much. “The funders had no role in study design, data collection and analysis, decision to publish, or preparation of the manuscript.”

If any authors received a salary from any of your funders, please state which authors and which funders.

**Response:** Thank you very much for time. “The authors received no specific funding for this work.”

The following is a point-to-point response to the reviewers’ comments.

Reviewer(s)' Comments to Author:

**Reviewer:**

**Major Issues/Concerns**

**Response to reviewer # 1:** Thank you very much. We appreciate you taking the time to offer us your comments and insights related to the paper. We found your feedback very constructive. We tried to be responsive to your concerns. We hope you find these revisions rise to your expectations.

Regarding Reviewer#1 major concerns, we make an explanation in detail as follow:

1. “Badly unclear written. Revise it as I revised for the first experiment.”

**Response:** Thank you very much for your kind revision. We have revised experiment 2^nd^, according to the reviewer suggestion. For instance “The first experiment comprised of five ratios of underground water to surface water including; 1:0 (U), 0:1 (S), 1:1 (T_3_), 1:2 (T_4_) and 1:3 (T_5_). While, the second experiment comprised of eight treatments including: 1:3 (T1), 2:2 (T2), 3:1 (T3), 1:3 {S: U (T4)}, 2:2 {S: U (T5)}, 3:1 {S: U (T6)}, 4:0 (T7) and 0:4 (T8).”

Please see our revised manuscript

2. Interactive effect of which factors? You should have at least two factors for an interaction effect.

**Response:** Many thanks. Basically in this sentence {“Furthermore, two-way ANOVAs revealed a significant main and interactive effect on dry matter accumulation at various growth stages treated with surface water in round irrigation scheme (*p* < 0.05)”} we present the results of two way ANOVA for which after coding the data in experiment 2^nd^, we consider Surface water (S) as a separate factor and underground water (U) as a separate factor while the S*U is their interactive factor, To be more clear and precise following is the ANOVA tables for Surface water, Underground water and their interaction for various growth stages, but in our manuscript we have linked these ANOVA tables results with their corresponding figures.

Please see figure 3

Table: Results of two-way ANOVAs for growth stages (stem, leaves and roots) of dry matter accumulation as dependent on surface water (*S*), underground water (*U*) and their interaction (S*×U*).

|  | Stem | | | Leaves | | Roots | |
| --- | --- | --- | --- | --- | --- | --- | --- |
| Source | df | F | P-value | F | P-value | F | P-value |
| S | 1 | 2.36 | 0.14 | 3.94 | **0.05*** | 3.06 | 0.09 |
| U | 1 | 0.73 | 0.40 | 0.03 | 0.95 | 0.08 | 0.77 |
| S×U | 1 | 6.23 | **0.02*** | 0.45 | 0.50 | 2.20 | 0.15 |

Table: Results of two-way ANOVAs for boll stages (stem, leaves and roots) of dry matter accumulation as dependent on surface water (*S*), underground water (*U*) and their interaction (S*×U*).

|  | Stem | | | Leaves | | Roots | |
| --- | --- | --- | --- | --- | --- | --- | --- |
| Source | df | F | P-value | F | P-value | F | P-value |
| S | 1 | 28.1 | **0.00*** | 12.9 | **0.00*** | 46.1 | **0.00*** |
| U | 1 | 3.30 | 0.08 | 0.00 | 0.94 | 0.31 | 0.58 |
| S×U | 1 | 297.8 | **0.00*** | 42.7 | **0.00*** | 115.8 | **0.00*** |

3. at various growth stages treated with surface water in round irrigation scheme (p < 0.05). Unclear! Revise it.

**Response:** Done, we have revised the whole sentence. i.e.

“Furthermore, the results of two-way ANOVAs revealed a significant main and interactive effect on dry matter accumulation treated with surface water at cotton plant growth, boll and boll opening stages (p < 0.05).”

**For instance, the highest leaves dry matter in round irrigation at various growth**

**What do you mean?**

**Response:** We apologize for the confusion. Basically in this study we used two different irrigation schemes i.e. (1) Different mixing ratio irrigation (2) Round irrigation. In experiment 1^st^ (different mixing ratio) we mix different ratios of surface water and underground water irrigation and after mixing we apply the irrigation water to the cotton field (Please see the attached pictures of the mixing ratios scheme), however in Experiment 2^nd^ (round irrigation) we apply the surface water and underground water directly to the cotton field without mixing it by using a water meter reader (attached is the picture), So to make it more understandable to the reader we use the term round
irrigation.

Kg per hectare? Per m2?.

**Response:** Yes, your consideration is right**.** The basic unit for cotton is yield in Kg/hm^2^, instead of kg/h.m^2^, Similar to our studies most of the paper used the same unit following are some of references

**Reference:**

Suping Wang, Xiaokun Li, Jianwei Lu , Juan Hong, Gang Chen , Xinxin Xue , Jifu Li , Yunxia Wei , Jialong Zou , Guangwen Liu. 2013. Effects of controlled-release urea application on the growth, yield and nitrogen recovery efficiency of cotton Agricultural Sciences 4 (2013) 33-38.

Zhongna Yang, Tarleton State University, Stephenville, TX Mark Yu Ashley Lovell. 2018. COTTON PRODUCTION IN XINJIANG PROVINCE AND ITS IMPACT TO COTTON MARKETS IN CHINA. 2018 Beltwide Cotton Conferences, San Antonio, TX, January 3-5, 2018.

Wang, G.; Feng, L.; Liu, L.; Zhang, Y.; Li, A.; Wang, Z.; Han, Y.; Li, Y.; Li, C.; Dong, H. Early Relay Intercropping of Short-Season Cotton Increases Lint Yield and Earliness by Improving the Yield Components and Boll Distribution under Wheat-Cotton Double Cropping. Agriculture 2021, 11, 1294

This is not a novel and strong conclusion, as your statement looks like a final result of a local report.

**Response:** Thank you for this direction, we have revised our conclusion in our revised manuscript. Following is the revised conclusion

“Overall, our findings provide useful information to current irrigation practices in water scarce regions. Alternatively, improving water use efficiency is a viable solution to the water scarcity. Therefore, surface water irrigation is recommended as an effective irrigation strategies to improve cotton yield and growth.”

Please see our revised manuscript with track changes

Patchy paragraphs.

You should write your introduction and all parts of your manuscript in a linked manner

**Response:** Thank you very much, we have potentially revised our introduction along with whole manuscript according to the reviewer suggestion. We hope our revised manuscript will meet the reviewer expectation. We strongly focus on paragraph linking and try to remove the patchy words as well as patchy paragraph.

Please see our revised manuscript

Most parts of your introduction talk about China and its problems, so it can not be interesting for audiences worldwide. It`s a locally issue!

**Response:** Totally agree. But in china the arid and semi-arid region (52 % land area) faces extreme shortage of irrigation water, especially in Xinjiang. As we have conducted our research study in Xinjiang therefore mostly we talk about Xinjaing water resources and cotton output. However in our introduction part we use some other countries references.

**2.2 Soil and Plants sampling**

**“Move before 2.4”.**

**Response:** Done. Please see our revised manuscript

“If RCBD is your mean!

RCBD: Randomized complete block design

CRD: Completely randomized design.

Be careful in writing the special phrase

**Response:** Extremely sorry for our careless job. Yes both of the experiment were Randomized complete block design (RCBD), we have carefully revised our both experimental design section.

Please see our revised track change manuscript.

How did you apply your treatments?

More details are needed on irrigation regimes.

Full detail should be presented

**Response:** Thank you very much. We applied our treatment by digging whole and supply underground water and surface water with a constant ratio by using a water ratio measuring instrument (please see the attached pictures of our experimental scheme), after mixing surface and underground water we mix the nutrients with the water and supply it to the cotton field through drip irrigation. We have added full information about irrigation regimes please see our revised manuscript.

Please see the attached pictures,

Revise them in a clear way.

**Response:** Thank you very much for this insightful comment. We have revised our experimental treatments. Please see our revised manuscript.

“The second experiment (round experiment) was a field randomized complete block design (RCBD), comprised of eight treatments including, 1:3 (T_1_), 2:2 (T_2_), 3:1 (T_3_), 1:3 {S:U (T_4_)}, 2:2 {S:U (T_5_)}, 3:1 {S:U (T_6_)}, 4:0 (T_7_) and 0:4 (T_8_).”

Which factors? You have just one factor!.

**Response:** We apologize for our mistake. We have corrected it in our revised manuscript. Please see our revised manuscript.

Why didn`t you talk about this in the materials and methods section! This is unclear for us! What do you mean?

**Response:** We are extremely sorry for the confusion. Now we have included the detailed information about round irrigation in our material and method section. Please see our revised experiment design section in our revised manuscript.

**Which methods? You had just one method based on what we saw in the materials and methods section. Your treatments are not different irrigation methods**

**Response:** Thank you very much. Here we talk about our two experimental schemes i.e. 1) Different mixing ratio experiment; 2) Round irrigation experiment. In the first experiment we use different mixing ratio by digging whole and supply constant ratio of surface and underground water into it. After that we mix the nutrients with the water and supply it to the cotton field. However in round irrigation we directly supply the required ratio of surface water and underground water into the cotton field without mixing it.

Just glance at your findings, then discuss them! You should **discuss your results**!

**Response:** Many thanks. We have completely revised our discussion section, please see our revised manuscript.

This could be a separate chapter without any relationship with your results. In all parts of discussion we should see traces of your work and your results.

**Response:** We have completely revised our discussion section please see our revised version of manuscript.

Please pay attention to what I said at the end of abstract.

Your conclusion is a bit better than abstract, but not satisfying.

**Response:** We apologize for the confusion. We have revised our conclusion section in our revised manuscript.

Update the references.

No 2022 article

Just one 2021 reference, which not an article.

**Response:** Done we have added some new references in our revised manuscript.
