# Supplementary material for: Effect of surface water and underground water drip irrigation on cotton growth and yield under two different irrigation schemes
Source: PLoS One. 2022 Oct 14;17(10):e0274574. doi: 10.1371/journal.pone.0274574 (PMC9565416; doi:10.1371/journal.pone.0274574)
Supplement: Attachment — Submitted filename: Response to Reviewers.doc [file pone.0274574.s008.doc]

**Listed responses to comments by the reviewers (**PONE-D-22-03631**)**

Dear Prof. Dr. Rafiq Islam
Academic Editor

PLOS ONE,

Many thanks for your letter. We greatly appreciate the constructive suggestions and comments on our MS entitled “Effect of surface water and underground water drip irrigation on Cotton growth & yield under two different irrigation schemes" from both yourself and the reviewers. All those comments are valuable and very helpful for us to improve our manuscript. We extend our great appreciation for taking the time and efforts to provide such insightful guidance. We have taken a complete consideration to all reviewers’ comments as well as those suggestions from the editor’s and have made the corrections one by one in the revised version of our manuscript. The changes in the revised MS are marked in track change model.

We sincerely hope the revised manuscript will be able to meet the requirement and will be finally accepted to publish on your journal of “PLOS ONE”. Of course, we are always available to provide ongoing changes to our manuscript if there is any further request either from you or from the reviews.

Below please find the revised manuscript and the responses. Again, thank you and all the reviewers for your kinder assistance and we are looking forward to hearing from you at your earliest convenience.

Best wishes

Many thanks again

On behalf of the all coauthors

Sincerely yours,

-------------------------------------------------------------------------------------------------------

The following is a point-to-point response to the reviewers’ comments.

**Comments from the editors and reviewers:**

**Response to the editors' comments**
Editor's note: 

The revised manuscript, in its current content, format, and interpretation, is unsuitable to accept for publication. The authors failed to address the concerns of the reviewer(s). I fact, none of the responses were realistic. It needs a total and thorough revision with appropriate statistical analysis, prestation of data in tables and figures, and interpretation of results.

**Response:** Extremely sorry for our careless job, we have now fully revised our manuscript. We hope that our revised manuscript will be raised to your expected level. Please see our revised manuscript

The abstract should be focused and concise to summarize the whole work. Introduction needs to be written based on global context with connecting paragraph links to each other with a mission and vision.
 
**Response:** Many thanks for your valuable comment. We have now revised our whole abstract

Please see our revised abstract section in our revised manuscript.

The methods and materials section need in-depth explanation about the experimental design, factors (fixed vs. random), cultural practices and fertilization, analysis of soil and plant samples, appropriate statistical analysis, and presentation of data in significant digits with interactions. Treatments should be separated as factors such as Underground vs. Surface water, Growth stages, soil depth, and time.

**Response:** Thank you very much, we have thoroughly revised our material and method section also we have incorporated some new statistics. We hope the current revised version will be able to meet your journal standard. .

Cleary explain the irrigation frequencies and timing, and volume, types and amount of fertilization, sampling of soil and plant samples, and methods of soil and plant analysis. The SOM cannot be determined by K2Cr2O7 oxidation method, it should be total organic carbon (TOC). When TOC multiplied with 1.724 then it will be SOM. Moreover, it should be “Perchloric acid.” Data need to present in mean + standard error of mean. Should add a section on water-use efficiency.

**Response:** Thank you very much. Please see our revised manuscript. We have incorporated all the editor comments point by point in our revised manuscript.

It would be good for the authors to consult a statistician to reorganize and analyse the data and interpret the results. Based on revised statistical data analysis, the results and discussion sections should be rewritten. The data presented in Table 5 to 8 were unrealistic, way too high for available NPK concentration during the season and at the end of the season. Reanalyze the samples or recalculate the data to verify the concentration. SOM contents do not really changed in a season!

**Response:** Thank you very much. Now we have completely revised our figures and tables. We hope our revised manuscript will be up to your raised expectation.

Conclusions should be concise and focused based on results with a suggestion

**Response:** Please see our revised conclusion section

The following is a point-to-point response to the reviewers’ comments.

Reviewer(s)' Comments to Author:

**Reviewer:**

**Major Issues/Concerns**

**Response to reviewer # 1:** Thank you very much. We appreciate you taking the time to offer us your comments and insights related to the paper. We found your feedback very constructive. We tried to be responsive to your concerns. We hope you find these revisions rise to your expectations.

Regarding Reviewer#1 major concerns, we make an explanation in detail as follow:

1. “Badly unclear written. Revise it as I revised for the first experiment.”

**Response:** Thank you very much for your kind revision. We have revised experiment 2^nd^, according to the reviewer suggestion. For instance “The first experiment comprised of five ratios of underground water to surface water including; 1:0 (U), 0:1 (S), 1:1 (T_3_), 1:4 (T_4_) and 1:3 (T_5_). While, the second experiment comprised of eight treatments including: 1:3 (T1), 2:2 (T2), 3:1 (T3), 1:3 {S: U (T4)}, 2:2 {S: U (T5)}, 3:1 {S: U (T6)}, 4:0 (T7) and 0:4 (T8).”

Please see our revised manuscript

2. Interactive effect of which factors? You should have at least two factors for an interaction effect.

**Response:** Many thanks. Basically in this sentence {“Furthermore, two-way ANOVAs revealed a significant main and interactive effect on dry matter accumulation at various growth stages treated with surface water in round irrigation scheme (p < 0.05)”} we present the results of two way ANOVA for which after coding the data in experiment 2^nd^, we consider Surface water (S) as a separate factor and underground water (U) as a separate factor while the S*U is their interactive factor, To be more clear and precise following is the ANOVA tables for Surface water, Underground water and their interaction for various growth stages, but in our manuscript we have linked these ANOVA tables results with their corresponding figures.

3. at various growth stages treated with surface water in round irrigation scheme (p < 0.05). Unclear! Revise it.

**Response:** Done, we have revised the whole sentence. i.e.

“Furthermore, the results of two-way ANOVAs revealed a significant main and interactive effect on dry matter accumulation treated with surface water at cotton plant growth, boll and boll opening stages (p < 0.05).”

**For instance, the highest leaves dry matter in round irrigation at various growth**

**What do you mean?**

**Response:** We apologize for the confusion. Basically in this study we used two different irrigation schemes i.e. (1) Different mixing ratio irrigation (2) Round irrigation. In experiment 1^st^ (different mixing ratio) we mix different ratios of surface water and underground water irrigation and after mixing we apply the irrigation water to the cotton field (Please see the attached pictures of the

mixing ratios scheme), however in Experiment 2^nd^ (round irrigation) we apply the surface water and underground water directly to the cotton field without mixing it by using a water meter reader (attached is the picture), So to make it more understandable to the reader we use the term round
irrigation.

Kg per hectare? Per m2?.

**Response:** Extremely sorry**.** The basic unit for cotton is yield in Kg h^-1^, instead of kg h^-1^, we have now revised it throughout in our revised manuscript

This is not a novel and strong conclusion, as your statement looks like a final result of a local report.

**Response:** Thank you for this direction, we have revised our conclusion in our revised manuscript.

Please see our revised manuscript with track changes

Patchy paragraphs.

You should write your introduction and all parts of your manuscript in a linked manner

**Response:** Thank you very much, we have potentially revised our introduction along with whole manuscript according to the reviewer suggestion. We hope our revised manuscript will meet the reviewer expectation. We strongly focus on paragraph linking and try to remove the patchy words as well as patchy paragraph.

Please see our revised manuscript

Most parts of your introduction talk about China and its problems, so it can not be interesting for audiences worldwide. It`s a locally issue!

**Response:** Totally agree. We have now removed most of the sentences which only focus on china we have now make it up to global context

**2.2 Soil and Plants sampling**

**“Move before 2.4”.**

**Response:** Done. Please see our revised manuscript

“If RCBD is your mean!

RCBD: Randomized complete block design

CRD: Completely randomized design.

Be careful in writing the special phrase

**Response:** Extremely sorry for our careless job. Yes both of the experiment were Randomized complete block design (RCBD), we have carefully revised our both experimental design section.

Please see our revised track change manuscript.

How did you apply your treatments?

More details are needed on irrigation regimes.

Full detail should be presented

**Response:** Thank you very much. We applied our treatment by digging whole and supply underground water and surface water with a constant ratio by using a water ratio measuring instrument (please see the attached pictures of our experimental scheme), after mixing surface and underground water we mix the nutrients with the water and supply it to the cotton field through drip irrigation. We have added full information about irrigation regimes please see our revised manuscript.

Please see the attached pictures,

Revise them in a clear way.

**Response:** Thank you very much for this insightful comment. We have revised our experimental treatments. Please see our revised manuscript.

Which factors? You have just one factor!.

**Response:** We apologize for our mistake. We have corrected it in our revised manuscript. Please see our revised manuscript.

Why didn`t you talk about this in the materials and methods section! This is unclear for us! What do you mean?

**Response:** We are extremely sorry for the confusion. Now we have included the detailed information about round irrigation in our material and method section. Please see our revised experiment design section in our revised manuscript.

**Which methods? You had just one method based on what we saw in the materials and methods section. Your treatments are not different irrigation methods**

**Response:** Thank you very much. Here we talk about our two experimental schemes i.e. 1) Different mixing ratio experiment; 2) Round irrigation experiment. In the first experiment we use different mixing ratio by digging whole and supply constant ratio of surface and underground water into it. After that we mix the nutrients with the water and supply it to the cotton field. However in round irrigation we directly supply the required ratio of surface water and underground water into the cotton field without mixing it.

Just glance at your findings, then discuss them! You should **discuss your results**!

**Response:** Many thanks. We have completely revised our discussion section, please see our revised manuscript.

This could be a separate chapter without any relationship with your results. In all parts of discussion we should see traces of your work and your results.

**Response:** We have completely revised our discussion section please see our revised version of manuscript.

Please pay attention to what I said at the end of abstract.

Your conclusion is a bit better than abstract, but not satisfying.

**Response:** We apologize for the confusion. We have revised our conclusion section in our revised manuscript.

Update the references.

No 2022 article

Just one 2021 reference, which not an article.

**Response:** Done we have added some new references in our revised manuscript.
