# Supplementary material for: Effect of surface water and underground water drip irrigation on cotton growth and yield under two different irrigation schemes
Source: PLoS One. 2022 Oct 14;17(10):e0274574. doi: 10.1371/journal.pone.0274574 (PMC9565416; doi:10.1371/journal.pone.0274574)
Supplement: Attachment — Submitted filename: Response to Reviewers.doc [file pone.0274574.s009.doc]

**Listed responses to comments by the reviewers (**PONE-D-22-03631**)**

Dear Professor Dr. Rafiq Islam

Academic Editor

PLOS ONE,

Many thanks for your letter. We greatly appreciate the constructive suggestions and comments on our MS entitled “Effect of surface water and underground water drip irrigation on Cotton growth & yield under two different irrigation schemes" from both yourself and the reviewers. All those comments are valuable and very helpful for us to improve our manuscript. We extend our great appreciation for taking the time and efforts to provide such insightful guidance. We have taken a complete consideration to all reviewers’ comments as well as those suggestions from the editor’s and have made the corrections one by one in the revised version of our manuscript. The changes in the revised MS are marked in track change model.

We sincerely hope the revised manuscript will be able to meet the requirement and will be finally accepted to publish on your journal of “PLOS ONE”. Of course, we are always available to provide ongoing changes to our manuscript if there is any further request either from you or from the reviews.

Below please find the revised manuscript and the responses. Again, thank you and all the reviewers for your kinder assistance and we are looking forward to hearing from you at your earliest convenience.

Best wishes

Many thanks again

On behalf of the all coauthors

Sincerely yours,

-------------------------------------------------------------------------------------------------------

The following is a point-to-point response to the reviewers’ comments.

**Comments from the editors and reviewers:**

**Response to the editors' comments**
Editor's note: 

While the revised manuscript quality has improved; it still needs a thorough editing for correcting grammatical mistakes and spelling to improve the quality of the research findings.

**Response:** Extremely sorry for our careless job, we have now fully revised our manuscript. Also we have revised our manuscript by a native English speaker for correcting grammatical mistakes and spellings. We hope that our revised manuscript will be raised to your expected level. Please see our revised manuscript

As abstract is the summary of the whole research, it needs an extensive review and editing. The authors should simplify the sentences for better flow of understanding.

 
**Response:** Many thanks for your valuable comment. We have thoroughly revised our abstract for better flow and understanding. We have our revised abstract section will be able to meet your criteria.

Please see our revised abstract section in our revised manuscript.

Moreover, the statistical analysis section is unclear to understand the variables, analysis and interpretation. Data presented in the Tables especially Table 4 is very confused, such as m^3^/667 h^-1^. It could be just m^3^/ha, and in the Materials and methods section, this could be explained as total volume of water applied for irrigating the cotton was … m^3^/ha. This needs to be very clearly explained in the methodology...

**Response:** Thank you very much. We have revised our material and method section according to the reviewer and editors suggestions along with tables and figures. We hope the current revised version will be able to meet your journal standard. .
